# Development of Functional *Casu Axedu* Cheese Enriched with Olive Leaf Phenolics and a Probiotic-Candidate *Lactiplantibacillus plantarum* Strain

**DOI:** 10.3390/foods15152694

**Published:** 2026-07-30

**Authors:** Maria Barbara Pisano, Carlo Ignazio Giovanni Tuberoso, Massimo Pes, Roberta Comunian, Margherita Addis, Luigi Chessa, Marco Caredda, Myriam Fiori, Giacomo Lai, Alessio Silvio Dedola, Valentina Masala, Dario Usai, Antonio Paba, Carlo Piga, Angela Carboni, Federica Mereu, Sofia Cosentino

**Affiliations:** 1 Department of Medical Sciences and Public Health, University of Cagliari, Strada Provinciale 8, 09042 Monserrato, Italy; barbara.pisano@unica.it; 2 Department of Life and Environmental Sciences, University of Cagliari, Strada Provinciale 8, 09042 Monserrato, Italy; tuberoso@unica.it (C.I.G.T.); valentina.masala2@unica.it (V.M.); dario.usai@unica.it (D.U.); 3 Agris Sardegna, Agricultural Research Agency of Sardinia, Loc. Bonassai, SS291 km 18.600, 07100 Sassari, Italy; mpes@agrisricerca.it (M.P.); rcomunian@agrisricerca.it (R.C.); maddis@agrisricerca.it (M.A.); lchessa@agrisricerca.it (L.C.); mcaredda@agrisricerca.it (M.C.); mfiori@agrisricerca.it (M.F.); gialai@agrisricerca.it (G.L.); adedola@agrisricerca.it (A.S.D.); apaba@agrisricerca.it (A.P.); cpiga@agrisricerca.it (C.P.); acarboni@agrisricerca.it (A.C.); 4 Independent Researcher, 09100 Caglisri, Italy; fedem8@hotmail.it

**Keywords:** olive leaf extract, *Lactiplantibacillus plantarum*, *Casu Axedu*, functional cheese, antioxidant capacity, nutritional properties

## Abstract

Functional fresh cheeses can serve as effective carriers of bioactive compounds and probiotic cultures. This study developed a *Casu Axedu* cheese fortified with olive leaf phenolics and a probiotic-candidate *Lactiplantibacillus plantarum* (LP) strain. Three formulations were produced from thermised sheep milk: a control (CA-I), a cheese with LP (CA-IL) and a cheese combining olive leaf extract (OLE; 20 mg GAE 100 g^−1^ milk) and LP (CA-IOL). In all experimental cheeses, inulin was included as a dietary fibre, and lactose hydrolysis was performed to improve product accessibility for lactose-intolerant consumers. Cheeses were stored at 3 ± 1 °C for 30 days and analysed for polyphenol profile, antioxidant capacity, physicochemical and nutritional characteristics, microbiological quality, sensory properties and consumer acceptance. OLE fortification approximately doubled the total polyphenols and produced a more than threefold increase in FRAP antioxidant capacity in CA-IOL, with a high percentage of the main olive leaf phenolics retained after 30 days. Composition, fatty acid profile, vitamin A/E and cholesterol contents were unaffected by OLE and LP addition; all formulations met “high in protein” and lactose-free label criteria (according to the guidelines issued by the Italian Ministry of Health DGISAN Communication No. 27673 of 7 July 2015), and the inulin-based “source of fibre” claim was supported by formulation and mass-balance calculation. Presumptive lactobacilli remained at high counts throughout storage in the LP-containing cheeses, and OLE neither impaired the lactic microflora nor promoted coliform growth. OLE addition increased perceived bitterness, astringency and olive-like notes in CA-IOL, moderately reducing liking scores relative to those of the control among the tested consumer panel (5.25 ± 1.7 at 30 d); mean liking nonetheless remained above the neutral point of the 9-point hedonic scale. Overall, combining inulin, olive leaf extract and a probiotic-candidate *L. plantarum* strain enabled the production of a *Casu Axedu* cheese with enhanced antioxidant properties and improved nutritional features while retaining acceptable sensory quality. This approach represents a promising strategy for the valorisation of olive by-products and the development of innovative sheep milk dairy products consistent with circular economy principles.

## 1. Introduction

Growing consumer interest in foods that combine traditional sensory quality with additional nutritional and health-promoting properties has stimulated the development of functional dairy products [1,2,3]. Cheese is a favourable matrix for such fortification because of its nutrient density, buffering capacity and ability to protect sensitive compounds during storage and gastrointestinal transit [4,5].

Among lactic acid bacteria (LAB), *Lactiplantibacillus plantarum* has attracted particular attention because of its remarkable metabolic versatility, adaptation to diverse ecological niches and technological robustness during food fermentations. *L. plantarum* is naturally associated with a wide range of fermented plant and dairy products and exhibits excellent tolerance to acid stress, osmotic pressure and other environmental conditions encountered during cheese manufacture and storage [6,7,8]. From a technological perspective, *L. plantarum* has been successfully employed as an adjunct culture in several cheese varieties, where it contributes to acidification, proteolysis and flavour development while maintaining high viable populations throughout storage. The proteolytic activity of selected strains may also promote the release of bioactive peptides with antioxidant or other biological properties, thereby further enhancing the functional potential of the cheese matrix [6,9]. Beyond its technological suitability, selected strains possess desirable functional characteristics, including resistance to gastrointestinal conditions, adhesion to intestinal epithelial cells, production of antimicrobial metabolites and modulation of host-associated microbial communities. These properties are highly strain-dependent and therefore, the selection and validation of individual strains remain essential prerequisites for their application as adjunct cultures in functional foods [7].

In parallel with the growing demand for functional foods, increasing attention has been devoted to the sustainable exploitation of agro-industrial by-products as sources of high-value bioactive compounds [10]. Olive cultivation is one of the most economically important agricultural activities throughout the Mediterranean basin, generating large quantities of leaves during annual pruning and olive harvesting. Traditionally regarded as agricultural waste, olive leaves are now recognised as a rich source of phenolic compounds with considerable biological and technological potential. Their valorisation is fully consistent with the principles of a circular economy and represents an opportunity to transform a low-value by-product into an ingredient suitable for food applications. Olive leaves (*Olea europaea*) are a well-documented source of phenolic compounds, including oleuropein, hydroxytyrosol and verbascoside, with antioxidant, anti-inflammatory and antimicrobial properties reported in the literature [11,12]. Because milk and most dairy products are naturally poor sources of phenolics, the direct addition of olive leaf extract (OLE) has been explored as a means of increasing the total phenolic content and antioxidant capacity of dairy matrices, generally without major changes to proximate composition or texture [13]. Depending on the dose and processing conditions, however, OLE addition can also increase bitterness and astringency and alter fermentation performance, indicating a need for matrix- and dose-specific optimisation [14,15]. The effect of OLE on the survival and functionality of specific probiotic-candidate strains such as *L. plantarum*, both in vitro and within the cheese matrix, has received comparatively little attention, and preliminary evidence from fermented table olives suggests that *L. plantarum* and olive-derived phenolics may interact favourably to enhance antioxidant content and limit spoilage microorganisms [16].

Despite these promising observations, relatively few studies have investigated the simultaneous incorporation of probiotic bacteria and olive-derived phenolic extracts into cheese. Most published investigations have evaluated either probiotic adjunct cultures or plant extracts independently, while information regarding their combined technological and functional effects remains limited [17]. In particular, little is known about how olive phenolics may influence the survival and metabolic activity of probiotic bacteria within cheese matrices or conversely, how bacterial metabolism may modify the phenolic profile of olive extracts during storage. Understanding these reciprocal interactions is essential for designing functional dairy products in which both microbial viability and bioactive compound stability are preserved.

Dietary fibre and lactose management are additional, complementary strategies in functional dairy development. Inulin is a widely used prebiotic fructan that increases dietary fibre content, generally without compromising cheese texture or sensory quality [18,19], while enzymatic lactose hydrolysis is a standard approach for producing lactose-reduced or lactose-free dairy products for lactose-intolerant consumers [20].

Fresh cheeses may provide particularly favourable matrices for this purpose because their relatively mild manufacturing conditions minimise the thermal degradation of heat-sensitive bioactive compounds while supporting the survival of adjunct bacterial cultures. Their high moisture content and limited ripening also allow the effects of added functional ingredients to be evaluated without the confounding influence of extensive biochemical changes occurring during prolonged maturation. *Casu Axedu* is a traditional Sardinian soft fresh cheese recognised as an Italian Traditional Agri-Food Product (PAT; MASAF, 2025) [21], typically produced from sheep and/or goat milk and characterised by high moisture content, delicate acidity and a distinctive sensory profile linked to the local dairy heritage [22]. It was selected as the model system for this study because its production involves only mild thermal treatment and a short, low-temperature fermentation without extended ripening, conditions that limit the thermal and enzymatic degradation of heat-sensitive phenolic compounds and are favourable to the survival of added lactic acid bacteria, in contrast to the conditions used for pressed or long-ripened cheese types. Unlike most fresh cheeses, coagulation and fermentation occur directly in the retail container, where the cheese remains throughout storage. Furthermore, the whey spontaneously released from the coagulum is retained within the package and forms an integral part of the product, being commonly consumed together with the cheese. Its strong regional identity and Traditional Agri-Food Product status also make it a relevant case study for functionally upgrading an existing traditional product without altering its identity. However, despite its cultural and technological significance, *Casu Axedu* has received comparatively little attention as a vehicle for functional food development. A recent multi-omics study reported favourable shifts in gut microbiota composition and activity in rats fed *Casu Axedu* [23], supporting further interest in its functional development.

The novelty of our work lies in the combined incorporation of inulin lactose hydrolysis, olive leaf extract and a probiotic-candidate *L. plantarum* strain (UNICA B26) within *Casu Axedu*, evaluated through an integrated technological, microbiological, chemical and sensory approach. Because inulin and lactase treatment were applied as a common functional base across all formulations, this design specifically allows for the additional effects of *L. plantarum* and olive leaf extract, rather than just the independent effects of inulin or lactose hydrolysis, to be assessed.

Therefore, the aim of the present study was to develop a functional *Casu Axedu* cheese enriched with olive leaf extract and a probiotic-candidate *L. plantarum* strain and to evaluate the effects of this combined fortification on (i) polyphenol content and antioxidant capacity, (ii) composition and selected technological properties, (iii) microbiological profile and lactobacilli viability, and (iv) sensory and consumer responses over 30 days of refrigerated storage.

## 2. Materials and Methods

### 2.1. Experimental Design

Three *Casu Axedu* formulations: CA-I (control), CA-IL (with the probiotic-candidate *Lactiplantibacillus plantarum* UNICA B26), and CA-IOL (with olive leaf extract (OLE) and *L. plantarum* UNICA B26), were produced from thermised sheep milk using a common inulin-enriched, lactose-hydrolysed base. Inulin and lactase treatment (Section 2.6) were applied to all three formulations to create a common functional base with enhanced dietary fibre content and reduced lactose content and to provide a uniform matrix in which the additional effects of *L. plantarum* and OLE could be evaluated. Consequently, CA-I is an inulin-enriched, lactose-hydrolysed control rather than a traditional *Casu Axedu*, and the present design evaluates the additional effects of *L. plantarum* and OLE within this already-functionalised matrix. Three independent cheesemaking trials were carried out on three different production days. In each trial, a fresh batch of sheep milk was used and divided into the three experimental formulations (CA-I, CA-IL, and CA-IOL), resulting in three independent replicates per formulation and a total of nine cheesemaking trials.

Cheeses were stored at 3 ± 1 °C and analysed after 1, 15 and 30 days of refrigerated storage. Different analytical tests (composition and nutritional indices, polyphenols/antioxidant capacity, syneresis, texture, colour, microbiological parameters, sensory and consumer tests) were performed according to the procedures described below.

### 2.2. Preparation of the Olive Leaves Extract

The polyphenol-enriched extract (OLE) was obtained from the olive leaves (*Olea europaea* variety *Bosana*) using an EtOH-H_2_O solution (60:40, *v*/*v*) at a solvent-to-plant ratio of 1:3.33 (*w*/*v*). The leaves were ground with an immersion blender and subsequently homogenised with an Ultra-turrax^®^ T50 (IKA-Werke GmbH, Staufen, Germany) high-performance dispersing instrument. The resulting mass was pressed to recover the liquid fraction which, after being coarsely filtered, was centrifuged for 15 min at 4000 rpm at 7 °C. The clear supernatant was concentrated under vacuum in a Rotavapor^®^ rotary evaporator (BUCHI, Newmarket, UK) to remove the ethanol. The resulting aqueous extract was filtered through a 0.22 µm membrane and plated on Plate Count Agar (PCA; Microbiol, Cagliari, Italy) to ensure microbiological suitability, then stored at −20 °C until use (no more than 20 days). The dry residue of OLE was determined in triplicate by drying the solution (2 g) in a thermostatic oven at 105 ± 1 °C for 8 h until constant weight. The final yield was found to be 8.96 ± 0.03 g of dry residue per 100 g of OLE.

### 2.3. Determination of Total Polyphenols Content (Folin-Ciocalteu Assay) in Olive Leaves Extract

The total polyphenols content (TP) of OLE was estimated using the Folin–Ciocalteu spectrophotometric assay on a Cary 50 UV–Vis spectrophotometer (Varian, Leinì, Torino, Italy). In brief, the absorbance was measured against a blank at 750 nm, using a calibration curve prepared with fresh gallic acid standard solution (25–500 mg L^−1^). Prior to analysis, OLE samples were diluted to 1:50 *v*/*v* with MeOH, and the TP results were expressed as grams of gallic acid equivalent (GAE) per 100 g of OLE [24].

### 2.4. Bacterial Strains

The *Lactiplantibacillus plantarum* (LP) strain used in this study, originally isolated from Sardinian ewe raw milk, is maintained in the UNICA-DSMSP Culture Collection under the accession code UNICA B26 (www.mbds.it, accessed on 14 May 2026), and is referred to throughout this manuscript as a probiotic-candidate strain, since formal demonstration of probiotic status under the relevant regulatory and scientific framework has not been performed. The strain was routinely propagated in De Man, Rogosa and Sharpe broth (MRS; Microbiol, Cagliari, Italy) at 30 °C under microaerophilic conditions. Its potential probiotic properties were previously described by Pisano et al. [25]; the strain has also previously been tested as a potential probiotic adjunct culture in caciotta cheese, where it showed good technological performance and the capacity to persist in large quantities within the cheese matrix throughout ripening [26].

The microbial starter culture used for *Casu Axedu* cheesemaking was comprised of four autochthonous bacterial strains belonging to the Agris-BNSS Culture Collection (www.mbds.it, accessed on 21 May 2026): *Lactococcus lactis* (BNSS 00542), *Lactococcus lactis* (BNSS 00576), *Lactococcus lactis* var. *diacetylactis* (BNSS 00763), and *Enterococcus faecium* (BNSS 00587). Each strain was individually cultivated in M17 medium (Microbiol, Cagliari, Italy), lyophilised, and combined to obtain a final inoculum suitable for 100 L of milk at a target concentration of 5 × 10^6^ CFU mL^−1^.

### 2.5. In Vitro Evaluation of the Effect of OLE on L. plantarum UNICA B26

The antibacterial activity of OLE against *L. plantarum* UNICA B26 was screened by broth microdilution to determine the minimum inhibitory concentration (MIC), following EFSA recommendations [27]. Serial twofold OLE dilutions (325–2.5 mg GAE 100 g^−1^, from an initial 1300 mg GAE 100 g^−1^) were prepared in Lactobacillus Susceptibility Medium (90% ISO-Sensitest broth, 10% MRS broth) in 96-well microtiter plates and inoculated with the strain to a final concentration of ~5 × 10^5^ CFU mL^−1^. Plates were incubated at 37 °C for 24–48 h under 5% CO_2_, and the MIC was recorded as the lowest OLE concentration showing no visible growth. All assays were performed in triplicate.

Tolerance of the strain to OLE was further assessed by inoculating MRS broth and UHT skimmed milk (7–8 log CFU mL^−1^) supplemented with OLE at 10 and 20 mg GAE 100 g^−1^, incubating for 24 h, and determining viable counts on MRS agar under microaerophilic conditions. These in vitro results demonstrate compatibility between the OLE and strain UNICA B26 under the tested conditions.

### 2.6. Casu Axedu Cheese Production

Raw whole sheep milk was obtained from the experimental farm of Agris Sardegna (Sassari, Italy) and processed as summarised in Figure S1. Milk was filtered, supplemented with inulin (Fibruline^®^ XL, Cosucra, Warcoing, Belgium; degree of polymerisation (DP) ≥ 20) at 30 g kg^−1^ milk, stirred at high speed using an Ultra-turrax ^®^ T50 disperser, thermised at 68 °C without holding time and immediately cooled to 36 °C, yielding thermised milk with inulin (TM-I).

TM-I was divided into three 10 kg batches: two used directly for CA-I and CA-IL production, and one fortified with OLE to a final concentration of 20 mg GAE 100 g^−1^ milk (TM-IO) for CA-IOL production. All batches were treated with commercial lactase (GODO YNL2, Danisco, Copenhagen, Denmark; 0.4 g kg^−1^ milk) and inoculated with the indigenous starter culture BNSS-CA (0.1 g kg^−1^ milk; ~6 log CFU mL^−1^). For CA-IL and CA-IOL, *L. plantarum* UNICA B26 (frozen concentrate) was added simultaneously with the starter at 0.50 mL kg^−1^ milk, to a final concentration of 6 log CFU mL^−1^.

Rennet (Chymostar™, Copenhagen, Denmark; 600 International Milk Clotting Unit—IMCU mL^−1^) was added at 43.2 IMCU kg^−1^ milk, and the coagulum was dispensed into 425 mL polypropylene containers (120 × 88 × 69 mm), held for 30–40 min for presamic coagulation, and fermented at 25 °C for 18 h. Coagulation was assessed empirically according to the traditional cheesemaking practice routinely adopted for *Casu Axedu* production. No appreciable differences among formulations were observed in coagulation time or coagulum formation during cheesemaking.

In each formulation, fermentation was monitored continuously using an eight-channel Liquiline CM448 pH system (Endress+Hauser, Gerlingen, Germany). Acidification curves (pH vs. time) were used to derive Vm (maximum acidification rate, mUpH min^−1^), Tm (time to Vm, min), and pHm (pH at Vm), according to the methods of Spinnler and Corrieu [28]; these parameters were used as indicators of lactic acid bacteria metabolic activity during fermentation. At the end of fermentation, the cheeses were cooled and stored at 3 ± 1 °C and analysed after 1, 15 and 30 days.

### 2.7. Compositional and Nutritional Characterisation of Milk and Cheese

#### 2.7.1. Extraction and Quali-Quantitative Determination of Target Polyphenolic Compounds in Cheese

Polyphenols were extracted from *Casu Axedu* according to the methods of Gil et al. [24]: 10 g of cheese were extracted with 10 mL of a EtOH:H_2_O mixture (60:40, *v*/*v*), homogenised with an Ultra-turrax^®^ T50 disperser for approximately 20 s at 14,000 rpm, sonicated for 10 min at 10 ± 2 °C and centrifuged for 15 min at 4000 rpm at 7 °C. The supernatant was transferred to 15 mL Falcon^®^ tubes and stored at −20 °C for 24 h to facilitate possible further precipitation or separation of the fatty phase on the surface. Samples were then diluted 1:5 (*v*/*v*) with 0.22 M H_3_PO_4_, filtered with 0.22 μm cellulose acetate filters and injected into a high-performance liquid chromatograph (HPLC). This extraction method was validated in agreement with the ICH guidance note [https://www.ema.europa.eu/en/documents/scientific-guideline/ich-q-2-r1-validation-analytical-procedures-text-methodology-step-5_en.pdf (accessed on 5 March 2026)] by determining calibration ranges, linearity, limits of detection (LOD), limits of quantification (LOQ), repeatability, recovery and selectivity for the target compounds in the cheese matrix (Table S1). The linearity (expressed by the coefficient of determination, r2) was evaluated by preparing standard mixtures at six different concentrations, and the calibration curves were plotted using the external standard method, correlating the peak area with the concentration by means of the least-squares method. The LODs and LOQs were calculated according to the equation LOD = 3.3 r/S and LOQ = 10 r/S, respectively (where r = standard deviation of the blank, and S = slope of the calibration curve). The repeatability of this method was evaluated using the relative standard deviation (% RSD) for the area under the peak after testing six injections of the same standard containing the target standards. The recovery rates were evaluated by spiking *Casu Axedu* cheese samples with two concentrations of the standard (see Table S1), and each spiked sample was analysed in triplicate. To evaluate selectivity, three blank matrices were used, and no interferences were observed in the same chromatographic window for either methodology.

Quali-quantitate analysis of the cheeses fortified with OLE was performed with a high-performance liquid chromatography–photodiode array detector (HPLC-PDA) technique following the method of Deluca et al. [29] and was specifically targeted to compounds belonging to the secoiridoids and flavonoids classes: luteolin glucoside was dosed at 360 nm, verbascoside at 313 nm, oleuropein at 280 nm, and hydroxytyrosol and hydroxytyrosol glucoside at 210 nm. Identification was based on comparison with pure standards, and for hydroxytyrosol glucoside, on UV–Vis spectral comparison with literature data [30,31,32,33,34,35,36].

#### 2.7.2. Milk

Raw sheep milk (RM), thermised milk with inulin (TM-I) and thermised milk with inulin and OLE (TM-IO) were analysed for fat, protein, casein, lactose and cryoscopy using a Milkoscan FT+ (Foss, Hillerød, Denmark) and for pH using a Crison Basic 20+ pH-meter (Crison Instruments S.A., Alella, Spain); dry matter was determined per ISO 6731 [37]. Inulin content in TM-I and TM-IO was not determined analytically; it was assumed to correspond to the added amount based on formulation and mass-balance calculation, supported by the observed increase in dry matter after fortification, matching the theoretical increase.

Total polyphenols (TP) in milk were determined by applying the method described by Velázquez Vázquez et al. [38] to extract and quantify them through the Folin–Ciocalteu spectrophotometric assay, using a Cary 1E spectrophotometer (Varian, Harbor City, CA, USA): absorbance was measured against a blank at 750 nm using a calibration curve prepared with fresh gallic acid standard solution (3–17 mg L^−1^), and results were expressed as grams of gallic acid equivalent (GAE) per 100 g of milk.

To estimate the net contribution of OLE, the total net polyphenol content (TPN) was calculated by subtracting the control sample without OLE from the content of total polyphenols determined in the OLE-fortified samples. Accordingly, TPN was calculated as TP(TM-IO) − TP(TM-I) for milk samples and as TP(CA-IOL) − TP(CA-IL) for cheese samples.

The total antioxidant capacity (TAC) was measured using the Ferric Reducing Ability of Plasma (FRAP) assay. Milk extraction was carried out according to the method of El-Fattah et al. [39], with some modifications. Briefly, skim milk was obtained by centrifuging whole milk at 3000 rpm for 30 min and carefully removing the upper cream layer. Deproteinised milk was prepared by adding 10 mL of trichloroacetic acid (TCA) aqueous solution (20% *w*/*w*) to skim milk. After 1 h at room temperature, the suspension was filtered through Whatman No. 40 filter paper, and the resulting filtrate was subjected to the FRAP assay, according to the procedure of Benzie and Strain [40]. Results were expressed as µmol L^−1^ of milk.

#### 2.7.3. Cheese

*Casu Axedu* samples (CA-I, CA-IL, CA-IOL) were analysed for moisture (ISO 5534) [41], fat [42], total nitrogen (TN) (ISO 8968-1) [43], protein content (TN × 6.38), and pH (Crison Basic 20+; Crison Instruments S.A., Alella, Spain).

Inulin content was estimated in the same manner as for milk, on a mass-balance basis; since *Casu Axedu* is marketed together with the whey phase released during manufacturing and storage, which remains within the final packaged product, substantial losses of inulin are not expected. Therefore, the inulin content of the final product was estimated from the amount added during formulation and mass-balance considerations.

The total polyphenol content (TP) was determined as described for milk, using a calibration curve in the range 33–130 mg L^−1^, and expressed as mg GAE per 100 g of cheese. Total net polyphenol content (TPN) was calculated as TP(CA-IOL) − TP(CA-IL), where CA-IL represented the corresponding control formulation without OLE. Total polyphenol recovery (TPR) was calculated as the ratio between TPN and the theoretical amount of polyphenols added through OLE (20 mg GAE 100 g^−1^ milk), expressed as a percentage.

The total antioxidant capacity (TAC) was measured using the FRAP assay according to the method of Yilmaz-Ersan et al. [44] and Benzie and Strain [40] and expressed as µmol kg^−1^ of cheese.

Sugars (lactose, galactose, glucose, tagatose) and myo-inositol were quantified using a gas chromatographic method, as described by Dedola et al. [45], on a Varian 3600 gas chromatograph equipped with a flame ionisation detector and a split–splitless injector (Varian, Harbor City, CA, USA).

The fatty acid profile in cheese samples was determined as described by Addis et al. [46].

Tocopherol (Vitamin E), total retinol (Vitamin A), and total cholesterol were determined using the reversed-phase HPLC method proposed by Panfili et al. [47] and Manzi et al. [48].

Individual free fatty acids (FFAs) were determined as previously described by Addis et al. [49].

Proteolysis was investigated by evaluating the evolution of proteolytic indices: soluble nitrogen (SN) at pH 4.6, soluble nitrogen in 12% trichloroacetic acid (SN-TCA) and soluble nitrogen in 10% phosphotungstic acid (SN-PTA), as described by Gripon et al. [50], and expressed as a percentage of total nitrogen.

Energy values were calculated using standard conversion factors to the quantified macronutrients, as follows: fat = 9 kcal g^−1^, protein = 4 kcal g^−1^, carbohydrates = 4 kcal g^−1^, and inulin = 1.9 kcal g^−1^. Total energy was expressed as kcal per 100 g of product.

The “high in protein” claim was evaluated according to Reg. (EC) No 1924/2006 [51] and Reg. (EU) No 1169/2011 [52], considering that at least 20% of the total energy value is provided by protein. The percentage of energy from protein was calculated as the ratio between protein-derived energy and total energy.

The “source of fibre” claim was assessed considering an inulin content ≥ 1.5 g per 100 kcal. The fibre contribution was attributed exclusively to the added inulin, and the ratio between inulin content and total energy was used to verify eligibility.

### 2.8. Texture Analysis

*Casu Axedu* samples were subjected to a penetration test using a TA-XT Plus texture analyser (Stable Micro Systems, Surrey, UK), equipped with a 20 mm diameter cylindrical probe, at 4 °C. Before texture analysis, the whey was completely removed by aspiration; then, texture measurements were performed with the cheese maintained in its original retail container.

The penetration depth was 20 mm from the sample surface, with pre-test, test, and post-test speeds of 1 mm s^−1^. Each sample was subjected to penetration directly on the free surface of the cheese.

During the test, a force–displacement curve was recorded, considering both positive and negative values, as reported by Salvatore et al. [53]. From the resulting curve, four parameters were determined: maximum positive force (*F*_max_, N; cheese hardness), positive area (N·mm; consistency), maximum negative force (N; adhesive force), and negative area (N·mm; adhesiveness).

Data were acquired and processed using Exponent software version 6.2.6.0 (Stable Micro Systems, Surrey, UK). The probe was cleaned after each measurement, and analyses were performed in quadruplicate for each sample.

### 2.9. Syneresis

Syneresis was determined using the differential weighing method. Each sample was weighed in its original packaging before whey removal. Whey was completely removed by aspiration, and the packaging, containing only the cheese, was reweighed. Syneresis (%) was calculated as the difference between initial and final sample weights, expressed as a percentage of the initial weight, using the following equation:

(1) $$Syneresis\;\left(\%\right)=\frac{Winitial\;\left(g\right)-Wfinal\;(g)}{\;Winitial\;(g)}\;\times \;100$$

where *Winitial* is the weight of the sample in its container before whey removal, and *Wfinal* is the weight of the container with cheese only after complete whey removal.

### 2.10. Colorimetric Analysis

Colour was measured on the free surface of cheese samples using a portable CR-400 colorimeter (Konica Minolta, Milan, Italy), following the calibration and measurement protocol of Aryana et al. [54]. The CIE L* a* b* colour space was used, where L* denotes lightness (0 = black, 100 = white), a* denotes chromaticity on the green (−) to red (+) axis, and b* denotes chromaticity on the blue (−) to yellow (+) axis.

Because the combination of L*, a*, and b* coordinates provide a more comprehensive description of chromatic variations than does the analysis of individual parameters, the total colour difference (ΔE) was also calculated according to the method of [39], using the following equation:

(2) $$\Delta E=\sqrt{\left[{\left(L_{2}-L_{1}\right)}^{2}+{\left(a_{2}-a_{1}\right)}^{2}+{\left(b_{2}-b_{1}\right)}^{2}\right]}$$

This parameter expresses the chromatic distance between cheese samples from the compared treatments (distance of CA-I vs. CA-IL; distance of CA-I vs. CA-IOL; distance of CA-IL vs. CA-IOL).

### 2.11. Microbiological Analyses

Microbial analyses were performed on thermised milk (TM) and *Casu Axedu* cheese (CA-I, CA-IL and CA-IOL) at 1, 15, and 30 days of storage at 3 ± 1 °C. For each sampling point, three biological samples were analysed for each experimental cheesemaking. For each experimental cheesemaking, samples were analysed in duplicate for enumeration of enterococci, mesophilic cocci, lactobacilli and coliforms. Tenfold serial dilutions were prepared in sterile peptone water (0.1% *w*/*v*), plated on selective media: Kanamycin Aesculin Azide (KAA, Biolife Italiana, Milano, Italy) agar for enterococci (42 °C, 48 h, aerobic), M17 agar (Microbiol, Cagliari, Italy) for mesophilic cocci (30 °C, 48 h, anaerobic), MRS agar (Difco, Milano, Italy) with 50 mg L^−1^ Vancomycin for lactobacilli (30 °C, 48 h, microaerophilic) and Violet Red Bile Agar (VRBA; VWR International, Milano, Italy) with 4-metilumbelliferil-β-D-glucuronide (MUG) for coliforms (37 °C, 24 h, aerobic) and incubated under recommended conditions, following standard protocols for sheep milk cheeses. For each biological sample, every tenfold serial dilution was plated in duplicate on two plates; therefore, for each treatment and dilution, six plates were analysed in total, and the mean value was used for calculations. Because the selective medium used for lactobacilli does not itself confirm the identity of *L. plantarum* UNICA B26, and strain-specific molecular confirmation was not performed on colonies recovered during storage, counts on this medium are referred to throughout the manuscript as presumptive lactobacilli, compatible with the inoculated strain. Bacterial counts were expressed as mean log CFU g^−1^ ± standard deviation.

### 2.12. Sensory and Consumer Analyses

The sensory profile of *Casu Axedu* (CA-I, CA-IL, CA-IOL) at 1, 15 and 30 days of storage (3 ± 1 °C) was assessed via quantitative descriptive analysis (QDA) [55] using a panel of 10 trained assessors (35–55 years, balanced by sex) following ten 1 h training sessions covering attribute elicitation, lexicon development and calibration (Table S2). Assessors were selected from a larger pool of internal staff belonging to a sensory-analysis group with more than 10 years of experience in applied sensory evaluation of sheep dairy products. Assessor selection and training were conducted with reference to the general principles of ISO 8586:2023 [56]. Prior to formal evaluation, panellists attended ten 1 h training sessions devoted to familiarisation with the product category, attribute elicitation, development of a consensual lexicon, definition of reference standards, and panel calibration of scale use. The final list of attributes, their sensory definitions, and the corresponding qualitative and quantitative reference standards used for panel calibration are reported in the Supplementary Materials (Table S1). Attribute intensity was scored on an unstructured 9-point scale (1 = very low, 9 = very high) against reference standards. Sample evaluation was replicated to assess panel discrimination ability, repeatability, and consensus.

Consumer acceptance was evaluated by a separate group of 60 adults (volunteers, 35–55 years old), balanced by sex, on cheeses at 1 and 30 days of storage at 3 ± 1 °C. Consumers were recruited among external subjects selected according to the required demographic criteria and their familiarity with, or propensity to consume, this product category. Overall liking was rated on a 9-point hedonic scale (1 = dislike extremely; 9 = like extremely; 5 = neither like nor dislike) [57]. In addition, a check-all-that-apply (CATA) questionnaire was administered according to the methods of Ares et al. [58]. From a predefined list of 22 terms, consumers were asked to select those considered applicable to each sample, including descriptors related to sensory properties, emotional responses and hedonic perception. Consumer sessions were conducted in accordance with the general principles for hedonic testing in a controlled sensory area, as described in ISO 11136:2014 [59].

Samples (~4.0 × 4.0 × 2.0 cm) were presented in odourless containers, with random three-digit codes, in a randomised balanced design [60] and in individual booths under controlled conditions, according to ISO 8589:2007, ISO 13299:2016 and ISO 22935-2:2023 [61,62,63]. Participants were informed about study procedures, allergens and data anonymisation, and participated voluntarily in accordance with IFST guidelines [64]; formal ethics committee approval was not required, as the study involved non-invasive sensory testing of conventional foods, with minimal risk.

### 2.13. Statistical Analysis

Data on the in vitro effect of OLE on *L. plantarum* UNICA B26 (Section 2.5) were analysed by one-way ANOVA, followed by Tukey’s post hoc test (GraphPad Prism v. 5.0, GraphPad Software, San Diego, CA, USA); a level of *p* < 0.05 was considered statistically significant.

Syneresis, texture, colorimetric, and compositional/nutritional data were analysed by two-way ANOVA, with formulation (FF: CA-I, CA-IL, CA-IOL) and storage time (S: 1, 15, 30 days) as fixed factors (Minitab 16 v. 1.0, Minitab Inc., State College, PA, USA), followed by Tukey’s HSD test, in which significant differences were found; the FF × S interaction was tested for all parameters and was not significant (*p* > 0.05 for all), so the main effects are presented separately.

Microbial counts were analysed by one-way ANOVA to assess differences in microbial concentrations among samples and time points (TM, 1, 15, 30-day cheese) within each *Casu Axedu* formulation and microbial group. This approach was selected to specifically evaluate the temporal evolution of each microbial group within individual formulations during shelf life, ensuring a direct and interpretable assessment of microbial dynamics. Tukey’s post hoc test was applied to identify specific significant differences, with statistical significance set at *p* ≤ 0.05. This method was considered appropriate for capturing treatment effects within each formulation, while avoiding unnecessary complexity that could arise from multifactorial models.

Sensory and consumer data were processed using Smart Sensory Box (v. 2.3.5, Smart Sensory Solutions, Sassari, Italy) and R Statistical Software (v. 4.3.1, R Foundation for Statistical Computing, Vienna, Austria). QDA data were analysed by analysis of variance (ANOVA), including formulation, storage time, and assessor as factors. Consumer overall liking data were analysed by ANOVA, including formulation, storage time, and consumer as factors. Assessor and consumer terms were included in the respective models as random factors to account for individual variability across evaluations. When significant effects were detected, mean comparisons were performed using Tukey’s post hoc test, with statistical significance set at *p* < 0.05. For clarity, the main tables report only the product-related effects. Differences in the frequency of CATA term selection were evaluated using Cochran’s Q test. Trained panel performance was considered satisfactory in terms of discrimination ability, repeatability, and agreement with the panel consensus, based on *p*-values, mean square error (MSE), and z-scores, respectively [65]. Accordingly, no assessors were excluded, and all sensory data were retained for subsequent analyses. To obtain an integrated overview of the sensory information, a multiple factor analysis (MFA) was performed on the sensory data blocks collected at the storage times common to all sensory approaches, namely QDA attribute intensities, CATA citation frequencies, and hedonic variables (trained-panel overall sensory quality and consumer overall liking). RV coefficients were calculated to assess the agreement among the different data blocks and with the global compromise configuration. MFA was used as an exploratory multivariate tool to visualise the relationships among descriptive attributes, consumer-derived terms, and hedonic responses.

## 3. Results and Discussion

### 3.1. Total Polyphenol Content of the Olive Leaf Extract

The main phenolic compounds identified in OLE by HPLC-PDA (Supplementary Materials Figure S2) belonged to the secoiridoid, flavone and simple phenol classes, consistent with the profile typically reported for olive leaf extracts [11,12], and included the five target compounds subsequently monitored in the fortified cheese (Section 3.3).

### 3.2. Evaluation of OLE Effect on L. plantarum UNICA B26

**Figure 1:**
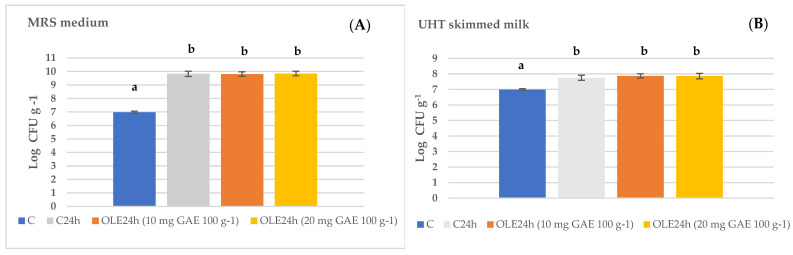
Effect of two different concentrations of OLE (10 mg and 20 mg GAE 100 g^−1^) on viability of the potential probiotic strain *L. plantarum* UNICA B26 in MRS (**A**) and in UHT skimmed milk (**B**) after 24 h incubation at 37 °C. Mean ± SD of three independent experiments. C = viable counts at time 0; C-24 h = viable counts detected after 24 h; OLE-24 h = viable counts detected after 24 h with the addition of OLE at 10 or 20 mg GAE g^−1^; different letters above the bars indicate significant differences (*p* < 0.05).

The preliminary screening carried out to analyse the antibacterial activity of the OLE showed that the *L. plantarum* UNICA B26 strain was not inhibited by the OLE at any of the concentrations tested, including the highest (MIC > 325 mg GAE 100 g^−1^); growth of the strain was slightly enhanced in the presence of OLE, increasing by about 2.5 log units after 24 h in MRS broth and by approximately 1 log unit in UHT skimmed milk, with no significant difference between cultures grown with or without the extract (*p* > 0.05, Figure 1). These results demonstrate compatibility between OLE and strain UNICA B26 under the tested in vitro conditions. This tolerance is consistent with the well-documented adaptability of *Lactobacillus* spp., and of *L. plantarum* in particular, to phenolic-rich environments [66,67,68].

### 3.3. Compositional and Nutritional Characterisation of Milk and Cheese

#### 3.3.1. Milk

Thermisation and fortification with inulin, with or without OLE, modified selected physicochemical and functional properties of sheep milk without significantly affecting its gross macro-nutrient composition (Table 1).

Indeed, pH, fat, protein, casein, lactose, and energy values did not differ significantly among RM, TM-I, and TM-IO (*p* > 0.05), suggesting that the treatments preserved the basic compositional characteristics of the milk matrix. By contrast, dry matter significantly increased in the fortified thermised samples, most likely due to the addition of inulin and other formulation solids (from OLE) (*p* < 0.05). This interpretation is supported by the addition of inulin (3%, *w*/*w*) to both TM-I and TM-IO samples. The lower fat-to-protein ratio detected in TM-I and TM-IO compared with that for RM should therefore be interpreted mainly as a consequence of formulation-related shifts in solids balance rather than as evidence of a biologically meaningful alteration in milk fat or protein fractions. Likewise, the more negative cryoscopic values of thermised fortified samples are coherent with the higher concentration of dissolved and dispersed components introduced by inulin supplementation and, in the case of TM-IO, by OLE addition.

The main differences were observed in relation to the functional profile of the milk. While inulin contributed to fibre enrichment, OLE fortification markedly increased both total polyphenol content and antioxidant capacity. TM-IO exhibited significantly higher TP and TAC values than did RM and TM-I, confirming the effectiveness of olive leaf extract as a source of bioactive antioxidant compounds in the dairy matrix. However, although OLE was added at 20 mg 100 g^−1^ of milk, the net polyphenol content recovered in TM-IO was only 11.4 mg GAE 100 g^−1^. This lower recovery may be explained by interactions between milk proteins and olive phenolic compounds, which are known to reduce the extractable and analytically measurable free phenolic fraction through non-covalent and, under some conditions, covalent binding mechanisms [69]. As highlighted by Velázquez Vázquez et al. [38], the protein- and fat-rich matrix of milk and in particular, the presence of proteins, can complicate the isolation, purification and analysis of phenolic compounds when added to milk.

Despite this incomplete analytical recovery, the strong increase in FRAP values observed in TM-IO indicates that the added phenolics retained substantial functional activity within the milk system. This is consistent with the view that protein–polyphenol interactions may limit phenolic extractability while still preserving antioxidant functionality in dairy matrices. Overall, these findings indicate that inulin and OLE played complementary roles, with inulin mainly acting as a fibre-enriching ingredient and OLE as the major contributor to the antioxidant and phenolic enhancement of thermised sheep milk [70].

#### 3.3.2. Quantitative Determination of Phenolic Compounds

The thermised sheep milk used for the *Casu Axedu* production, fortified with OLE (TP = 20 mg GAE 100 g^−1^ milk, TM-IO), was investigated to evaluate the presence of phenolic compounds immediately after the fortification and throughout their evolution during storage at +3 °C. after 1, 15 and 30 days. Table 2 and Figure S2 report the amount of the five target phenolic compounds, i.e., luteolin-7-*O*-glucoside (L-glu), verbascoside (Verb), hydroxytyrosol (HTyr), hydroxytyrosol glucoside (HTyr-glu), and oleuropein (Olup), dosed via the HPLC-PDA method. These five target phenolic compounds were selected because they represented the main phenolic classes (simply phenols, flavones, secoiridoids and lignans) in OLE extract (Figure S2), and their dosage, in the chromatograms at the specific wavelengths, was optimal due to the lack of interfering compounds.

**Figure 2:**
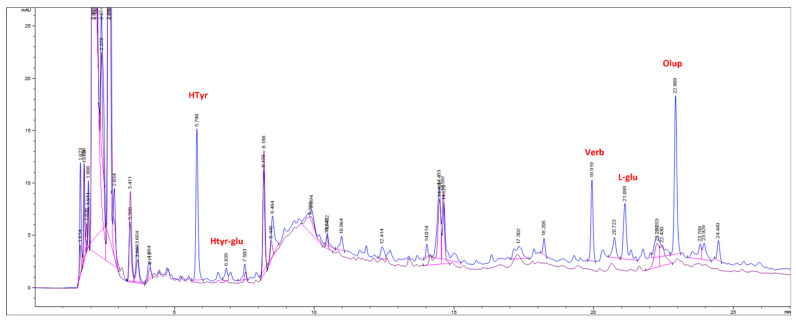
Chromatogram at λ = 280 nm of blank (CA-IL, *Casu Axedu* cheese fortified with inulin—DP ≥ 20—and *Lactiplantibacillus plantarum* UNICA B26; line in purple) and fortified *Casu Axedu* at 30 days (CA-IOL, *Casu Axedu* cheese fortified with inulin—DP ≥ 20—with OLE (TP = 20 mg GAE 100 g^−1^ of milk) and with *Lactiplantibacillus plantarum* UNICA B26; line in blue). L-glu: luteolin-7-*O*-glucoside, Verb: verbascoside, HTyr: hydroxytyrosol, HTyr-glu: hydroxytyrosol glucoside, and Olup: oleuropein.

Figure 2 reports the chromatograms at λ = 280 nm of blank (CA-IL) and fortified *Casu Axedu* at 30 days (CA-IOL), showing the separation of the dosed phenolic compounds.

The most abundant compounds in the thermised sheep milk used for the *Casu Axedu* production after the addition of OLE (TM-IO) were oleuropein and luteolin-7-*O*-glucoside (18.31 ± 3.40 and 8.59 ± 1.08 mg/100 g, respectively). After 1 day, some changes in the relative amounts were observed, and during the next 15 and 30 days, some of them revealed a significant trend (Figure S3). During storage, oleuropein and hydroxytyrosol glucoside decreased, with a smaller decrease between days 15 and 30 than between day 0 and day 15; hydroxytyrosol increased progressively (from 0.59 ± 0.08 to 1.71 ± 0.09 mg 100 g^−1^), while luteolin-7-*O*-glucoside and verbascoside remained essentially stable. The increase in hydroxytyrosol may be associated with the hydrolysis of oleuropein and related secoiridoids during storage. Such bioconversion has recently been demonstrated during lactic acid bacteria fermentation of olive leaves and has been attributed to the β-glucosidase activity of selected LAB strains [71]. Despite the absence of investigation into enzymatic activity in the present study, a comparable mechanism, in conjunction with the acidic nature of the cheese matrix, may have been responsible for the concurrent decline in oleuropein and rise in hydroxytyrosol observed during storage. Conversely, luteolin-7-*O*-glucoside and verbascoside demonstrated only minor variations, suggesting enhanced stability during storage.

Overall, 76% of the main polyphenolic compounds initially present were retained after 30 days, indicating that fortification at 20 mg GAE 100 g^−1^ of milk was sufficient to enrich *Casu Axedu* with the typical olive leaf polyphenols and that this enrichment was largely preserved over 30 days of refrigerated storage.

Olive leaf extracts have been the subject of significant research due to their high content of oleuropein, hydroxytyrosol, and related secoiridoids. These natural antioxidants have been extensively studied in the context of dairy systems, where their ability to enhance oxidative stability, functional properties, and product shelf life has been demonstrated [15,72,73,74,75]. Overall, polyphenol addition in dairy systems requires controlled dosing to balance technological functionality and sensory quality [15,73,74]. Beyond the addition of yoghurt, factors such as timing (i.e., the milk vs. curd ratio for cheese production) and formulation context are crucial in determining phenolic retention, textural quality, and colour, while ensuring the preservation of microbial viability and safety [76,77,78]. In the context of dairy matrices, olive-derived polyphenols such as oleuropein and hydroxytyrosol have been observed to contribute to antioxidant activity. However, the stability and functionality of these polyphenols are contingent upon processing and matrix interactions [15,75,79,80]. The prolongation of shelf life can be attributed to the antimicrobial properties that hinder the growth of harmful microbes or the onset of spoilage in dairy systems, as evidenced by several studies [15,72,73]. However, these effects are contingent on formulation and matrix and necessitate dose optimisation to circumvent sensory concerns. Oleuropein (OLE) has been demonstrated to enhance microbial stability in milk at controlled concentrations (1.44–3.6 mg oleuropein mL^−1^), without compromising sensory quality [72,81,82]. The antimicrobial and antioxidative properties of components derived from olive leaves or pomace have been demonstrated in cheese and milk [83,84]. As demonstrated in the cited literature, the utilisation of olive leaves and olive pomace as polyphenol-rich ingredients has the potential to enhance the circular economy’s principles of waste reduction and the valorisation of agro-industrial by-products.

#### 3.3.3. *Casu Axedu*

The formulation factor significantly affected several compositional parameters of *Casu Axedu* (Table 3). Since all formulations shared the same inulin-enriched, lactose-hydrolysed cheese matrix, the present study was designed to evaluate the additional effects of olive leaf extract and *Lactiplantibacillus plantarum* UNICA B26, rather than the individual effects of inulin supplementation or lactose hydrolysis. The slight but significant pH decrease observed from CA-I to CA-IOL (*p* < 0.05), in the presence of *L. plantarum* and OLE, remained within the usual range reported for *Casu Axedu* [23], suggesting that functional enrichment was achieved without compromising the typicity of this traditional product. A potential pH-lowering effect associated with OLE was also reported by other authors and may be linked to a promoting effect of OLE on the metabolic activity and growth of lactic acid bacteria [14,15,85]. Compared with CA-I and CA-IL, CA-IOL showed slightly lower dry matter and fat contents (*p* < 0.05), whereas protein levels remained comparable among formulations. This suggests that the combined inclusion of OLE and the selected *L. plantarum* strain did not impair the protein density of the product, and it is reasonable to assume that the observed differences in dry matter are mainly driven by small variations in fat content and moisture associated with the OLE addition.

The data reported in the literature are not always consistent regarding the effect of polyphenols on the water-holding capacity of the casein network [86,87]. However, some studies on the addition of OLE to dairy products showed a slight increase in the water-binding capacity of the protein network, resulting in higher moisture content in the final product [15,88]. The lower fat content observed in CA-IOL may be partly attributed to a diluting effect associated with its higher moisture content. Nevertheless, some authors suggested that the lower fat values in the samples containing OLE were due to a reduction in the analytical recovery of fat because of the formation of complexes between polyphenols and lipids, thereby reducing the detectable fat fraction [89]. Total energy (≈111–117 kcal 100 g^−1^) and the proportion of energy derived from protein (≈22% of kcal) were not affected by the formulation factor, indicating that these fortified fresh cheese products retained the nutritional profile and the “high protein” claim, according to EU Regulation 1924/2006.

Based on the amount of inulin added during formulation (30 g kg^−1^ milk), the theoretical inulin content of all formulations (CA-I, CA-IL and CA-IOL) was approximately 3%. As *Casu Axedu* is marketed together with the whey released during manufacturing and storage, substantial losses of inulin in the final product are not expected. Consequently, eligibility for the “source of fibre” claim (≥1.5 g inulin 100 kcal^−1^) was assessed on a formulation basis and mass-balance considerations rather than on the direct analytical determination of inulin in cheese.

The most pronounced effects of formulation were detected for total polyphenols content (TP) and total antioxidant capacity (TAC). CA-I and CA-IL displayed comparable TP levels (≈12 mg GAE 100 g^−1^), indicating that inulin and *L. plantarum* alone did not significantly contribute to the phenolic profile. Conversely, CA-IOL showed a marked increase in TP (23.8 mg GAE 100 g^−1^) and a more than threefold rise in TAC (1903 vs. 560–606 µmol kg ^−1^), demonstrating that OLE represented the main driver of the functional upgrading of *Casu Axedu* in terms of TP and TAC. These findings are consistent with previous observations on the use of olive leaf-derived ingredients in dairy matrices, where substantial improvements in antioxidant activity have been reported without detrimental effects on product identity or quality [15].

Despite the standardised addition of OLE at 20 mg GAE 100 g^−1^ of milk, the total net polyphenol content attributable to the extract (TPN ≈ 11 mg GAE 100 g^−1^) and the corresponding recovery (TPR ≈ 50–60%) indicated that only about half to two-thirds of the added phenolics were analytically recovered as extractable compounds. This behaviour reflects the well-documented propensity of milk proteins, particularly caseins and whey proteins, to interact with plant polyphenols through non-covalent and, in some conditions, covalent mechanisms, thereby reducing the measurable free phenolic fraction in fortified dairy products [38,90,91]. Nevertheless, the marked increase in TAC values observed in CA-IOL suggests that a considerable proportion of OLE polyphenols remained functionally active within the cheese matrix, even if partially sequestered in protein–polyphenol complexes and thus, less accessible to routine spectrophotometric assays. This interpretation is in line with recent reviews indicating that protein binding may limit polyphenol extractability, while maintaining, or even modulating, their antioxidant functionality and stability in dairy-fermented systems [90].

Regardless of the formulation, storage at 3 ± 1 °C up to 30 days had limited effects on the studied parameters. Most compositional traits (pH, dry matter, fat, energy and energy from protein) and TAC were not significantly influenced by storage time, indicating satisfactory stability of the fortified *Casu Axedu* during refrigerated shelf life. Protein content showed a modest but significant decrease at 30 days (*p* < 0.05), which can be ascribed to proteolysis and moisture redistribution commonly observed in fresh and short-ripened cheeses, but remained compatible with the maintenance of the “high protein” status of the products.

Total polyphenol content (TP) in *Casu Axedu* increased progressively during refrigerated storage, from 13.4 mg GAE 100 g^−1^ at day 1 to 16.4 and 18.5 mg GAE 100 g^−1^ at days 15 and 30, respectively, whereas total antioxidant capacity (TAC, FRAP method) remained statistically unchanged, with values close to 1000 µmol L^−1^ throughout the 30-day period. This divergence suggests that storage enhances the Folin–Ciocalteu response without producing a corresponding, significant gain in reducing power, as captured by FRAP. The simultaneous increase in TP and decrease in protein content in *Casu Axedu* at 30 days are compatible with proteolysis and suggest that the apparent increase in “phenolic” content may be due to matrix evolution. On the one hand, casein degradation into smaller peptides is likely to weaken protein–polyphenol interactions and to improve the extractability of olive leaf phenolics, thereby increasing the fraction detectable by the extraction protocol. On the other hand, the formation of peptides and exposure of amino acid residues with reducing groups (e.g., tyrosine, tryptophan, cysteine) may contribute directly to the Folin–Ciocalteu signal, which is known to respond to a broad range of non-phenolic reducing substances, in addition to polyphenols [92].

The observation that the total net polyphenol content (TPN) attributed to the olive leaf extract and its recovery do not change significantly over storage, despite the increase in TP, supports the hypothesis that the higher TP values at later times reflect a combination of enhanced phenolic release and growing interference from proteolysis products in the Folin–Ciocalteu assay rather than a true accumulation of polyphenols. The absence of a parallel, significant effect of storage on TAC is consistent with this view: if part of the additional Folin–Ciocalteu response is due to non-phenolic proteolysis products, only limited changes in FRAP would be expected. Overall, refrigerated storage of olive-leaf-enriched *Casu Axedu* appears to preserve antioxidant capacity while modulating the analytically detectable TP through matrix dynamics and proteolysis-related interference in the Folin–Ciocalteu assay.

Total sugars and individual monosaccharides (galactose, glucose), disaccharides (lactose), minor carbohydrates (tagatose) and myo-inositol showed no significant differences among CA-I, CA-IL and CA-IOL, nor across storage times (*p* > 0.05, Table 4). This suggests that the addition of inulin, *L. plantarum* and olive leaf extract did not alter the residual carbohydrate profile of the cheese, which remained low, as expected for an acid-coagulated, short-ripened product such as *Casu Axedu*. The absence of significant changes over 30 days indicates that any carbohydrate metabolism associated with the adjunct cultures occurred mainly during early fermentation, without further measurable depletion of residual sugars during refrigerated storage. The addition of commercial lactase (GODO YNL2, Danisco) to the initial TM-I and TM-IO milk allowed an almost complete hydrolysis of lactose, making it possible to obtain a lactose-free *Casu Axedu* cheese. In fact, according to the guidelines issued by the Italian Ministry of Health on the labelling of lactose in dairy products, a cheese with a lactose content lower than 0.1 g/100 g may bear the claim “lactose-free” (Italian Republic, Ministry of Health, DGISAN Communication No. 27673 of 7 July 2015).

Similarly, the fatty acid profile was remarkably stable across formulations and storage time (*p* > 0.05, Table 5). The proportions of saturated (≈70% of FAME), unsaturated, trans, monounsaturated and polyunsaturated fatty acids, as well as the PUFA n-6, PUFA n-3, and their ratio, did not differ among CA-I, CA-IL and CA-IOL and were unaffected by storage time. This stability is consistent with a product derived from the same raw milk and processing conditions, where fortification occurs primarily through inulin and phenolic extract rather than via lipid manipulation, and confirms that the fortified *Casu Axedu* cheeses preserve the typical fatty acid pattern of the traditional product.

Within the fat-soluble fraction, only modest formulation effects were observed. Vitamin A and cholesterol content were slightly lower in CA-IOL than in CA-I and CA-IL (*p* < 0.05, Table 5), whereas vitamin E content showed no significant differences. The slightly reduced vitamin A and cholesterol levels in CA-IOL are consistent with its marginally lower fat content reported previously, but the magnitude of these changes is limited and does not alter the overall nutritional positioning of the product. Importantly, storage did not significantly affect vitamins or cholesterol, supporting the oxidative stability of the fat fraction under the adopted conditions.

Free fatty acid (FFA) levels, whether expressed as short-, medium- or long-chain FFAs, as well as total FFA, did not differ between formulations and remained stable during storage (*p* > 0.05, Table S3). This indicates that neither the presence of *L. plantarum* nor the inclusion of OLE promoted lipolysis beyond the low levels expected in a fresh, acid-coagulated cheese and that the functional *Casu Axedu* formulations are not prone to lipid hydrolysis during 30 days of storage under refrigerated conditions. Such stability in FFA content is consistent with the maintenance of sensory quality and supports the suitability of *Casu Axedu* as a carrier for added bioactive compounds, without triggering excessive lipolytic activity. By contrast, proteolysis indices showed a clear effect of storage time, but not of formulation. SN/TN, TCA-SN/TN and PTA-SN/TN all increased significantly from day 1 to day 30, reflecting the progressive breakdown of caseins into water-soluble peptides and low-molecular-mass nitrogenous compounds, as typically observed in fresh and short-ripened cheeses. The absence of differences among CA-I, CA-IL and CA-IOL suggests that inulin, *L. plantarum* and OLE do not substantially modify the global proteolytic pattern of *Casu Axedu*, which remains primarily driven by the intrinsic enzyme system of milk and the lactic microbiota associated with the traditional process.

### 3.4. Texture, Whey Release, and Colour Parameters

The effects of formulation factor (FF) and refrigerated storage (S) at 3 °C for 1, 15, and 30 days on texture parameters, whey release, and colour attributes of *Casu Axedu* cheeses are reported in Table 6.

#### 3.4.1. Texture Parameters

Formulation significantly affected several penetration parameters, whereas storage time exerted a more limited effect. *F*_max_ (maximum penetration force, an indicator of gel hardness) was significantly lower in CA-IOL (1.6 N) than in CA-I (2.2 N) and CA-IL (2.1 N) (*p* < 0.05). The very similar values observed for CA-I and CA-IL indicate that *Lactiplantibacillus plantarum* UNICA B26 did not significantly affect the physical structure of the cheese matrix under the specific conditions tested. Therefore, the differences observed in CA-IOL are more likely associated with the incorporation of OLE, which may have contributed to changes in the structural organisation of the casein gel, resulting in a weaker and less cohesive protein network. A possible explanation could be related to the interactions between milk proteins and phenolic compounds. Protein–polyphenol interactions have been widely described in dairy systems [93,94] and may influence the organisation and physicochemical properties of the casein network during coagulation [95]. The presence of phenolic compounds could potentially interfere with these processes by altering casein conformation or affecting mineral equilibria within the micelle, potentially limiting casein–casein interactions and resulting in a less compact gel structure [96]. These differences in penetration behaviour are consistent with the proposed interpretation and are reflected in the penetration profiles (Figure S3), where CA-I and CA-IL exhibit very similar curve shapes and peak forces, whereas CA-IOL shows a clearly lower maximum peak and a slightly smoother profile during penetration, indicating that a lower force was required to deform the gel network.

Storage time influenced *F*_max_, which decreased significantly from 2.2 N at day 1 to 1.7 N at day 30 (*p* < 0.05), likely reflecting progressive structural modifications of the protein matrix associated with proteolysis increase (Table 6, *p* < 0.05). This trend would be consistent with the proteolysis indices reported in Table S3, showing increased water-soluble nitrogen and peptide fractions over the storage period, supporting the observed softening of the gel network, in line with mechanisms described in the literature [97]. The penetration curves, reported in Figure S4, also illustrate this evolution, with slightly lower peak forces and smoother profiles at later storage stages, indicating a progressive weakening of the casein network. Although the positive area, representing the total mechanical work required for probe penetration into the matrix, was not significantly affected by formulation or storage time, CA-IOL showed a consistent trend toward lower values (23 N·mm) compared with CA-I (26 N·mm) and CA-IL (25 N·mm), which would indicate a slightly lower amount of energy required to deform the gel structure. Negative *F*_max_ and negative area, which describe the force and energy required to detach the probe from the gel, were significantly affected by formulation factor. CA-IOL exhibited a lower absolute negative *F*_max_ (−0.41 N) compared with that of CA-I (−0.57 N, *p* < 0.05), which tended to be lower than that of CA-IL (−0.53 N, *p* = 0.08), suggesting reduced adhesive interactions. A similar trend was observed for negative area, which was significantly lower in CA-IOL (−4.4 N·mm) than in CA-I (−6.4 N·mm) and CA-IL (−5.9 N·mm) (*p* < 0.05). These results are consistent with the hypothesis that polyphenols may interfere with casein–casein interactions, thereby contributing to modifications in the protein network structure. However, the observed reduction in adhesiveness may not solely reflect changes in internal cohesion but could also be associated with alterations in surface properties and gel–probe interactions. This interpretation is in agreement with the structural effects previously reported for milk protein–polyphenol systems [93,94], particularly regarding the modulation of protein–protein interactions and casein network organisation. Consistently, the withdrawal phase of the penetration curves (Figure S3) shows a lower-magnitude negative peak for CA-IOL, further supporting the reduced adhesive behaviour of this formulation. Storage time had a significant effect on the negative area, with the highest absolute value observed at 15 days (−6.1 N·mm), which was significantly higher than at day 1 (−5.0 N·mm, *p* < 0.05), while the value at day 30 (−5.6 N·mm) did not differ significantly from that of either day 1 or day 15. This trend may reflect general phenomena observed in cheese systems, such as transient hydration and rearrangement within the casein matrix during early storage [98], followed by progressive proteolytic weakening at later stages, which could also affect textural properties, including adhesiveness, as generally described in studies on cheese matrix evolution and ripening dynamics [97].

#### 3.4.2. Whey Release

Syneresis was significantly affected by formulation (*p* < 0.05), but not by storage time. Among the formulations, CA-IOL exhibited the lowest whey release (23%), whereas CA-I and CA-IL showed higher values (30%). The reduced whey release in CA-IOL is consistent with the higher moisture content observed in CA-IOL (Table 3) and may reflect a lower degree of gel contraction, possibly due to OLE-induced changes in the protein network. This interpretation aligns with reported structural effects of protein–polyphenol interactions, which can alter network organisation and water retention in casein matrices [90]. Similar results were reported by other authors for yoghurts enriched with polyphenols derived from olive leaves, showing that this interaction between milk proteins (caseins in particular) and polyphenols led to the formation of a protein network with enhanced water-binding capacity [15,88].

#### 3.4.3. Colour Parameters

The colourimetric parameters were moderately affected by formulation factor and remained largely stable during storage. Lightness (L*) showed very similar values among CA-I, CA-IL and CA-IOL (90-91), indicating that the different formulations did not substantially modify the overall brightness of the cheese matrix. Significant differences were instead observed for the chromatic coordinates. CA-IOL showed a slightly higher a* value (−2.2) than did CA-I (−2.8) and CA-IL (−2.6) (*p* < 0.05), indicating a modest shift toward less negative values and therefore, a reduction in the green hue. The b* values tended to be higher in CA-IOL (8.7) than in CA-I (8.1; *p* = 0.08) and were significantly higher than those of CA-IL (7.8; *p* < 0.05), indicating a moderate increase in the yellow component. These variations are consistent with the chromatic contribution of phenolic compounds from OLE and their interactions with the cheese matrix, as previously reported for dairy systems enriched with plant extracts [78]. Overall colour differences between formulations were quantified using the CIELAB colour distance parameter ΔE, which quantifies the overall colour difference between two samples by combining variations in L*, a* and b* coordinates. In the present study, ΔE values ranged from 0.6 (CA-I vs. CA-IL) to 1.5–1.6 for comparisons involving CA-IOL. These values indicate very limited colour differences between formulations. In fact, ΔE values below 1 are generally considered imperceptible, whereas values between 1 and 3 correspond to only minor differences that are barely perceptible to the human eye [99]. Therefore, although statistically significant variations in a* and b* coordinates were detected, the overall visual differences among cheeses would likely remain minimal. These results are consistent with the relatively low total phenolic content of the experimental cheeses (0.20 mg GAE g^−1^), which was markedly lower than that reported by Zandona et al. [78] for cheeses enriched with higher levels of OLE (up to 2.21 mg GAE g^−1^), where ΔE values exceeding 3 indicated clearly perceptible colour changes. Consequently, the modest ΔE values observed in the present study confirm that the incorporation of OLE at the tested concentration produced only slight chromatic modifications without substantially altering the visual appearance of the product.

### 3.5. Microbiological Parameters

**Figure 3:**
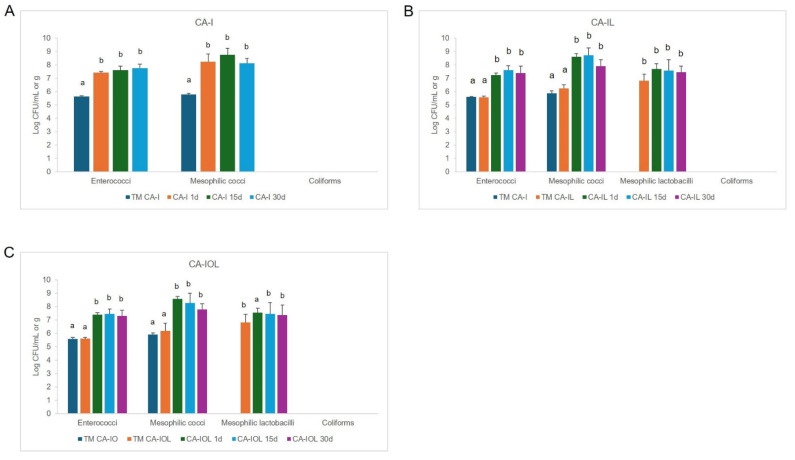
Evolution of key microbial groups in *Casu Axedu* manufactured with: (**A**) inulin (CA-I), (**B**) inulin and *Lactiplantibacillus plantarum* UNICAB26 (CA-IL), and (**C**) inulin, *L. plantarum* UNICAB26, and olive-derived extract (CA-IOL). Enterococci, mesophilic cocci, mesophilic lactobacilli, and coliforms were enumerated by plate count in thermised milk (TM) and in cheeses at 1, 15, and 30 days of storage; results are expressed as mean log CFU g^−1^. For each microbial group within each technological treatment, columns sharing at least one common letter do not differ significantly according to one-way ANOVA, followed by Tukey’s post hoc test (*p* ≤ 0.05).

The microbiological profile of *Casu Axedu* is shown in Figure 3, reporting log CFU g^−1^ counts of enterococci, mesophilic cocci, mesophilic lactobacilli, and coliforms at 1, 15, and 30 days of refrigerated storage at 3 ± 1 °C for CA-I, CA-IL, and CA-IOL.

**Figure 4:**
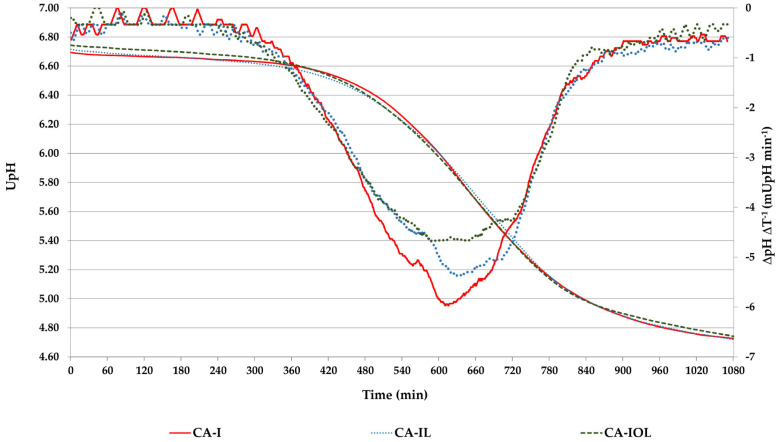
Evolution of pH and acidification rate in *Casu Axedu* cheese: CA-I, *Casu Axedu* cheese fortified with inulin (DP ≥ 20) (control); CA-IL, *Casu Axedu* cheese fortified with inulin (DP ≥ 20) and *Lactiplantibacillus plantarum* UNICA B26; CA-IOL, *Casu Axedu* cheese fortified with inulin (DP ≥ 20), OLE (TP = 20 mg GAE 100 g^−1^ of milk) and *Lactiplantibacillus plantarum* UNICA B26. ΔpH ΔT^−1^ represents the rate of pH change over time (first derivative), expressed as thousandths of pH units per minute.

In control CA-I, enterococci reached 7–8 log CFU g^−1^ at all storage times, with no significant differences (*p* ≤ 0.05) among 1, 15, and 30 d samples; mesophilic cocci showed similarly high levels, while coliforms remained below the detection limit (<1 log CFU g^−1^), indicating good hygienic quality. In thermised milk (TM) and in the inulin-enriched control cheese (CA-I) at day 1, the lactobacilli levels were below the detection limit and were therefore not enumerated in subsequent control samples. CA-IL supplementation with *L. plantarum* UNICA B26 maintained enterococci and mesophilic cocci at about 7–8 log CFU/g across shelf life (no significant differences, *p* ≤ 0.05), with coliforms undetectable and mesophilic lactobacilli counts of approximately 7 log CFU g^−1^ from day 1 onwards. Given the absence of detectable autochthonous lactobacilli in CA-I under the same selective conditions, these counts can reasonably be attributed to the intentionally added *L. plantarum* UNICA B26, indicating successful integration and persistence of the probiotic-candidate strain without interfering with the native starter lactic microbiota. In CA-IOL, enterococci and mesophilic cocci levels mirrored those observed in CA-IL (about 7–8 CFU g^−1^, *p* ≤ 0.05), and lactobacilli counts remained around 7 log CFU g^−1^ from day 1 onwards. This could indicate that the mesophilic lactobacilli population in both CA-IL and CA-IOL was essentially represented by the added probiotic-candidate strain. Refrigerated storage for 30 days induced only limited changes in the microbial populations. These findings demonstrate that inulin supplementation supports the stable development of desirable enterococci and mesophilic cocci in *Casu Axedu*, reaching technologically relevant populations (7–8 log CFU g^−1^) throughout 30 days of storage, in agreement with previous reports showing that inulin can sustain high lactic bacterial loads and improve microbial activity during cheese storage [100,101]. *L. plantarum* UNICA B26 addition (CA-IL) integrates effectively into the cheese microbiota, maintaining viability above the level generally considered adequate for delivering probiotic benefits (>7 log CFU g^−1^), and without interfering with the starter lactic populations, in line with data for dairy-derived *L. plantarum* strains [101,102]. The successful survival of *L. plantarum* UNICA B26 throughout refrigerated storage, despite the presence of olive phenolic compounds, is in agreement with the observations of Ngamsomchat et al. [9], who demonstrated that *L. plantarum* maintained high viable counts during the manufacture and storage of probiotic chèvre cheese, confirming the suitability of this species as an adjunct culture in fresh dairy products. Likewise, de Oliveira et al. [6] reported that *L. plantarum* CNPC003 remained metabolically active during the refrigerated storage of fresh cheese, contributing to product quality without compromising technological performance. Finally, the pH and ΔpH/Δt profiles (Figure 4) exhibited similar trends across the three *Casu Axedu* formulations, with largely overlapping curves and comparable minimum pH values. These observations were corroborated by the kinetic parameters derived from the acidification curves (Table S4), for which no significant differences (*p* > 0.05) were detected in the maximum acidification rate (Vm), the time required to reach Vm (Tm), or the corresponding pH value (pHm). Specifically, Vm ranged from -6.6 to -5.8 mUpH min^−1^, Tm from 631 to 678 min, and pHm from 5.64 to 5.81. Overall, these findings indicate that the addition of *L. plantarum* UNICA B26 and OLE did not affect the acidification performance of the starter culture under the conditions tested. From a technological perspective, this indicates that the incorporation of OLE and *Lactiplantibacillus plantarum* UNICA B26 did not interfere with the acidification kinetics during *Casu Axedu* manufacture, allowing the traditional cheesemaking procedure to be maintained without technological adjustments.

### 3.6. Sensory and Consumer Results

The sensory profile obtained by QDA (Table 7) confirmed that the formulation factor was the main source of variation for the descriptive attributes most closely linked to functional enrichment. In the QDA model, the individual assessor did not significantly affect attribute ratings, confirming satisfactory panel consistency; therefore, interpretation focused on formulation and storage-time effects. As reported in Table 7, the addition of *L. plantarum* alone had a limited impact on the sensory identity of *Casu Axedu*: CA-I and CA-IL did not differ significantly in overall sensory quality (6.03 and 5.77, respectively) and showed comparable values for colour, bitter taste, fresh milk aroma, olive oil aroma, astringency and off-flavour. The only marked difference attributable to the probiotic adjunct was a higher sour taste intensity in CA-IL than in CA-I (4.83 vs. 4.43; *p* = 0.020), consistent with the metabolic contribution of the adjunct culture, without evidence of sensory defects.

By contrast, the cheese containing both *L. plantarum* and olive leaf extract (CA-IOL) was clearly discriminated by trained assessors. Compared with CA-I and CA-IL, CA-IOL showed higher perceived colour intensity (3.31 vs. 2.71–2.83; *p* < 0.001), bitter taste (3.57 vs. 2.42–2.41; *p* < 0.001), olive oil aroma (3.30 vs. 2.18–2.24; *p* < 0.001), astringency (4.16 vs. 3.59–3.75; *p* < 0.001) and off-flavour (2.57 vs. 1.57–1.82; *p* < 0.001), together with lower fresh milk aroma (3.29 vs. 3.58–3.62; *p* < 0.001) and sweet taste (3.24 vs. 3.52 for CA-I; *p* = 0.02). These changes resulted in a lower trained-panel overall sensory quality for CA-IOL (4.84) than for CA-I and CA-IL (6.03 and 5.77; *p* < 0.001). The sensory impact of CA-IOL is therefore mainly attributable to OLE, rather than to the probiotic adjunct, and reflects the typical sensory contribution of olive-derived phenolics.

This interpretation is coherent with the chemical and physicochemical results reported above. CA-IOL was the formulation with the highest total polyphenol content and antioxidant capacity and contained olive-leaf phenolics such as oleuropein, luteolin-7-*O*-glucoside, verbascoside and hydroxytyrosol. The same phenolic enrichment that improved the functional profile of the cheese also plausibly explains the higher bitterness, astringency and olive-like notes perceived by the trained panel. The higher sensory colour intensity of CA-IOL was consistent with the instrumental colour data, which showed a modest increase in a* and b* coordinates in the OLE-fortified cheese, although the total colour differences remained small. Conversely, the instrumental softening, lower adhesiveness and reduced syneresis observed for CA-IOL were not paralleled by significant sensory differences in cohesiveness or watery perception. This suggests that the OLE-induced structural modifications of the protein network, although analytically measurable, remained less relevant to perception than did taste and aroma changes. This discrepancy may also be partly explained by the different sample preparation used for the analyses: for logistical and organisational reasons, sensory tests were conducted on portioned samples, and the resulting disruption of the curd structure may have affected texture perception by the assessors, whereas structural analyses were performed on the whole curd.

Storage time exerted a secondary but significant effect on selected sensory attributes. Colour, sour taste and bitter taste changed during refrigerated storage, while trained panel overall sensory quality evaluations decreased from 5.87 at day 1 to 5.22 at day 30 (*p* = 0.020). The increase in bitterness over storage (from 2.59 to 3.05; *p* < 0.001) may be related to the progressive evolution of the protein matrix and phenolic fraction during storage. In particular, the increase in proteolysis indices reported for the same samples (Table S3) may have contributed to taste evolution, whereas the absence of significant formulation effects on free fatty acids indicates that the sensory changes were not driven by lipolysis. At the same time, acidic odour, acidic aroma, sheep milk aroma, watery perception and cohesiveness remained stable during storage, indicating that the main sensory identity of the fresh cheese was preserved throughout shelf life.

The consumer test, performed after 1 and 30 days of storage, confirmed the trend observed by trained assessors. In the liking analysis, consumer liking was a significant source of variability, as commonly observed in hedonic studies, reflecting inter-individual differences in liking responses. Nevertheless, a clear formulation effect emerged. As reported in Table 8, at day 1, consumer liking was highest for CA-I (6.33), intermediate for CA-IL (6.13) and lowest for CA-IOL (5.61; *p* = 0.0237). After 30 days, CA-IL reached the highest liking score (6.49), followed by CA-I (6.11), whereas CA-IOL remained significantly less liked (5.26; *p* < 0.001). CATA responses clarified the drivers of this preference pattern. Consumers selected bitter taste much more frequently for CA-IOL than for CA-I and CA-IL both at day 1 (0.366 vs. 0.049–0.049; *p* < 0.001) and at day 30 (0.645 vs. 0.097–0.129; *p* < 0.001). At day 30, CA-IOL was also more frequently associated with olive oil aroma (0.258 vs. 0.065–0.097; *p* = 0.006), and less frequently with positive or identity-related terms such as balanced taste (0.097 vs. 0.419–0.452; *p* = 0.001), traditional (0.032 vs. 0.194–0.226; *p* = 0.032) and natural (0.194 vs. 0.419–0.419; *p* = 0.047). These results indicate that consumers detected the OLE-related sensory shift and that this perception reduced product acceptability when the bitter/astringent component became dominant. However, although significantly lower, the mean liking score of the CA-IOL sample remained above 5 on the 9-point hedonic scale, corresponding to the anchor “neither like nor dislike”, thus indicating moderate acceptability within the tested consumer group rather than unequivocal rejection. Consistently, consumers did not significantly associate CA-IOL with abnormal tastes or odours at either storage time, despite the higher off-flavour score detected by trained assessors. This distinction is important because it indicates that the lower liking of CA-IOL was not due to spoilage-related defects, but rather to the sensory expression of the phenolic enrichment. This interpretation is supported by the microbiological results: coliforms were absent, while enterococci, mesophilic cocci and *L. plantarum* B26 persisted at technologically relevant levels during storage. Therefore, the lower acceptability of CA-IOL should be interpreted as a formulation issue related to OLE dosage and sensory balance, rather than as a loss of microbiological quality. Overall, the present results suggest that further formulation optimisation is advisable to reduce bitterness and astringency while preserving the functional benefits associated with OLE fortification.

The results are also consistent with the literature summarized for similar cheeses. Previous studies reported that *L. plantarum* can modulate aroma development by increasing and diversifying volatile metabolites in fresh cheeses, with effects that are strongly strain- and matrix-dependent [103]. In the present study, *L. plantarum* B26 did not negatively affect the sensory profile of *Casu Axedu* and CA-IL showed the highest consumer liking after 30 days, in agreement with reports describing neutral or positive acceptability of *L. plantarum*-supplemented fresh or soft cheeses [104,105]. On the other hand, the lack of a clear sensory or instrumental textural change in CA-IL differs from results obtained with exopolysaccharide-producing *L. plantarum* strains in low-fat Akawi cheese, where hardness and adhesiveness were reduced [106]. In this work, softening and lower adhesiveness were observed mainly in CA-IOL and were more plausibly related to protein–polyphenol interactions than to the probiotic adjunct alone. Overall, the comparison with the work of previous authors confirms that probiotic addition can be compatible with sensory quality, whereas polyphenol fortification requires careful dose optimisation to balance functional improvement and consumer acceptance.

The multiple factor analysis provided an integrated confirmation of these results (Table S4 and Figure S6). The high RV coefficient between QDA and CATA (RV = 0.9005) indicated strong agreement between the descriptive sensory space generated by trained assessors and the terms selected by consumers. The hedonic dataset (EDO), including the overall sensory quality judgements of trained assessors and consumer liking, was also strongly correlated with CATA (RV = 0.9031) and, to a slightly lower extent, with QDA (RV = 0.7934). All three data blocks showed high correlation with the global compromise configuration (RV = 0.9361–0.9798). In the MFA map, bitter taste, astringency/astringent mouthfeel, olive oil aroma, off-flavour, and unpleasant/annoying descriptors were grouped in the same sensory region, opposite to consumer liking, overall sensory quality, balanced taste, naturalness, traditionality, and fresh milk aroma (fresh m Ar). This multivariate pattern, together with the univariate QDA and CATA results, supports the interpretation that the sensory shift observed for the OLE-containing cheese (CA-IOL) was mainly driven by bitterness, astringency, and olive-related notes, which were negatively associated with hedonic response. The individual factor map further showed that the CA-IOL samples were projected closer to this negative sensory region, whereas CA-I and CA-IL were located nearer to the positive hedonic descriptors.

## 4. Conclusions

This study shows that *Casu Axedu* can be developed into a fibre-enriched, lactose-reduced functional cheese combining olive leaf phenolics and a probiotic-candidate *Lactiplantibacillus plantarum* strain (UNICA B26), without altering the main technological identity of this traditional Sardinian fresh cheese. To our knowledge, this is the first study evaluating the combined use of OLE and an autochthonous *L. plantarum* strain in *Casu Axedu* cheese, thereby extending previous research on functional dairy products within a traditional sheep milk matrix.

OLE fortification was an effective and sustainable way to increase the phenolic content and antioxidant capacity of the cheese: total polyphenols approximately doubled and FRAP antioxidant capacity increased more than threefold relative to the inulin-only control, confirming the strong contribution of OLE to the functional profile of the product, retained after 30 days of refrigerated storage. These improvements were achieved without altering gross composition, fatty acid profile, or vitamin A/E and cholesterol content beyond minor formulation-related shifts and while maintaining compliance with the “high in protein” nutrition claim. Moreover, inulin and lactose hydrolysis provided a common nutritional background enabling compliance with “source of fibre” and “lactose free” claims, the latter in accordance with the Italian regulatory framework.

OLE and *L. plantarum* did not impair the main lactic microbiota of *Casu Axedu*, and coliforms remained undetectable in all formulations; these results indicate that OLE did not promote coliform growth, supporting the microbiological stability and hygienic quality of the product. Presumptive lactobacilli, consistent with the inoculated strain, persisted at concentrations generally associated with probiotic delivery throughout storage, although strain-specific molecular confirmation was not performed; the cheese can therefore be considered a suitable carrier for strain delivery.

Sensory and consumer evaluation showed that OLE fortification increased bitterness, astringency and olive-related notes and moderately reduced liking relative to the control among the tested consumer group. However, liking scores remained above the neutral point of the hedonic scale, indicating that CA-IOL was moderately acceptable, while formulation optimization, particularly of the OLE dose, may further reduce bitterness and astringency and improve acceptance.

Overall, the combined use of inulin, lactose hydrolysis, olive leaf extract and *L. plantarum* UNICA B26 appears as a feasible strategy to enhance the antioxidant potential, probiotic delivery and nutritional profile of *Casu Axedu*. From an industrial perspective, the use of olive leaf extract is consistent with circular economy strategies, contributing to the valorisation of olive by-products.

Despite these promising results, some limitations should be more clearly acknowledged. The lack of a traditional (non-inulin, non-lactase-hydrolysed) *Casu Axedu* control prevents direct comparison with the conventional product; inulin retention in the final cheese was assessed by mass balance rather than direct analysis; the identity of lactobacilli during storage was not confirmed at the strain level; and consumer results are representative of the tested panel rather than of the wider market. Moreover, the storage period was restricted to 30 days, corresponding to the typical *Casu Axedu* shelf life. Longer storage periods may influence probiotic survival, the stability of phenolic compounds, lipid oxidation, texture development and sensory quality. It could be of interest the use of OLE and the *L. plantarum* UNICA B26 strain in different cheese types with longer ripening periods. Finally, although *L. plantarum* UNICA B26 has previously demonstrated probiotic potential, in this study, no gastrointestinal survival, colonisation potential or in vivo health effects after cheese consumption were investigated. Future research could address these aspects by extending storage studies, confirming strain identity at the molecular level, evaluating the bioaccessibility and bioavailability of olive phenolics released from the cheese matrix, and investigating the biological activity of the fortified product through in vitro gastrointestinal digestion models and human intervention studies.

## Figures and Tables

**Table 1:** Physicochemical and macro-compositional parameters of milk.

	RM ^1^	TM-I ^2^	TM-IO ^3^	SEM ^7^	*p*-Value ^8^
pH (UpH)	6.51	6.56	6.62	0.07	ns
Dry matters (%)	16.4 ^b^	18.8 ^a^	18.5 ^a^	0.2	*
Fat (%)	6.3	6.2	6.1	0.1	ns
Protein (%)	5.2	5.2	5.1	0.1	ns
Casein (%)	3.8	4.1	4.1	0.1	ns
Fat/protein (-)	1.220 ^a^	1.197 ^b^	1.186 ^b^	0.005	*
Cryoscopy (°C)	−0.52 ^a^	−0.70 ^b^	−0.72 ^b^	0.02	*
Lactose (%)	4.1	3.9	3.9	0.2	ns
TP (mg GAE 100 g^−1^) ^4^	4.4 ^b^	4.4 ^b^	15.8 ^b^	0.3	*
TPN (mg GAE 100 g^−1^) ^5^			11.4		-
TAC (µmol L^−1^ of milk) ^6^	478 ^b^	502 ^b^	1499 ^a^	30	*

^1^ RM, raw sheep milk; ^2^ TM-I, (control) thermised sheep milk, fortified with inulin (DP ≥ 20); ^3^ TM-IO, thermised sheep milk, fortified with inulin (DP ≥ 20) and OLE (TP = 20 mg GAE 100 g^−1^ of milk); ^4^ TP, total polyphenol content; ^5^ TPN, total net polyphenol content calculated as TP (TM-IO)–TP (TM-I). ^6^ TAC, total antioxidant capacity (FRAP method); ^7^ SEM, standard error of the mean; ^8^ *p*-value, ns, no significant difference (*p* > 0.05); * *p* < 0.05; different superscript letters within rows indicate significant differences.

**Table 2:** Quantitative determination of target phenolic compounds (mg 100 g^−1^, mean ± SD, *n* = 3).

Target Phenolic Compounds	TM-IO ^1^	1 Day	15 Days	30 Days
L-glu	8.59 ± 1.08 ^a^	8.10 ± 0.40 ^a^	9.65 ± 0.57 ^a^	9.30 ± 1.45 ^a^
Verb	0.87 ± 0.07 ^a^	1.03 ± 0.03 ^b^	1.05 ± 0.04 ^b^	0.97 ± 0.09 ^ab^
HTyr	0.59 ± 0.08 ^a^	1.21 ± 0.28 ^b^	1.75 ± 0.26 ^bc^	1.71 ± 0.09 ^c^
HTyr-glu ^2^	0.32 ± 0.04 ^a^	0.30 ± 0.06 ^a^	0.14 ± 0.02 ^b^	0.11 ± 0.09 ^b^
Olup	18.31 ± 3.40 ^a^	13.57 ± 2.99 ^ab^	11.81 ± 3.52 ^ab^	9.69 ± 3.57 ^b^

^1^ TM-IO, thermised sheep milk, fortified with inulin (DP ≥ 20) and OLE (TP = 20 mg GAE 100 g^−1^ of milk); ^2^ dosed as HTyr equivalents. L-glu: luteolin-7-*O*-glucoside, Verb: verbascoside, HTyr: hydroxytyrosol, HTyr-glu: hydroxytyrosol glucoside, and Olup: oleuropein. Mean values within a line with different letters are significantly different at *p* < 0.05.

**Table 3:** Chemical–nutritional and functional parameters of *Casu Axedu* enriched with inulin and olive leaf extract and inoculated with *Lactiplantibacillus plantarum* UNICA B26 during storage.

	Formulation Factor			Storage Time at 3 ± 1 °C				*p*-Value ^11^		
	CA-I ^1^	CA-IL ^2^	CA-IOL ^3^	1 Day	15 Days	30 Days	SEM ^10^	FF ^12^	S ^13^	FF × S ^14^
pH (UpH)	4.42 ^a^	4.36 ^ab^	4.32 ^b^	4.39	4.36	4.35	0.04	*	ns	ns
Dry matter (%)	22.4 ^a^	22.0 ^a^	21.2 ^b^	21.9	22.1	21.6	0.4	*	ns	ns
Fat (%)	8.5 ^a^	8.5 ^a^	7.6 ^b^	8.4	8.3	8.0	0.3	*	ns	ns
Protein (%)	6.5	6.4	6.4	6.8 ^A^	6.5 ^A^	6.0 ^B^	0.2	ns	*	ns
TP (mg GAE 100 g^−1^) ^4^	12.0 ^b^	12.4 ^b^	23.8 ^a^	13.4 ^C^	16.4 ^B^	18.5 ^A^	0.8	*	*	ns
TPN (mg GAE 100 g^−1^) ^5^			11.4	12.1	12.3	10.8	0.9	-	ns	ns
TPR (%) ^6^			57	61	62	49	4	-	ns	ns
TAC (µmol kg^−1^ of cheese) ^7^	560 ^b^	606 ^b^	1903 ^a^	1012	1068	989	70	*	ns	ns
Energy from protein (%) ^8^	22.2	22.0	21.8	22.2	22.1	21.8	0.4	ns	ns	ns
Energy (kcal 100 g^−1^) ^9^	117	115	111	120	115	109	4	ns	ns	ns

^1^ CA-I, *Casu Axedu* cheese fortified with inulin (DP ≥ 20) (control); ^2^ CA-IL, *Casu Axedu* cheese fortified with inulin (DP ≥ 20) and *Lactiplantibacillus plantarum* (UNICA B26); ^3^ CA-IOL, *Casu Axedu* cheese fortified with inulin (DP ≥ 20) and OLE (TP = 20 mg GAE 100 g^−1^ of milk) and *Lactiplantibacillus plantarum* (UNICA B26); ^4^ TP, total polyphenol content; ^5^ TPN, total net polyphenol content calculated as TP (CA-IOL)–TP (CA-IL); ^6^ TPR, total polyphenol recovery, calculated as TPN in CA-IOL sample/TP added in milk (20 mg GAE 100 g^−1^ of milk) × 100; ^7^ TAC, total antioxidant capacity (FRAP method); ^8^ product “high in protein”: at least 20% of total calories from protein (Reg. (EC) 1924/2006 and Reg. (EU) 116/2010); ^9^ Energy is determined taking into account the calories provided by each nutrient (fat: 9 kcal g^−1^; protein: 4 kcal g^−1^; carbohydrates: 4 kcal g^−1^; inulin: 1.9 kcal g^−1^); ^10^ SEM, standard error of the mean; ^11^ *p*-value, ns, no significant difference (*p* > 0.05); * *p* < 0.05; ^12^ FF, formulation factor; ^13^ S, storage time factor at 3 ± 1 °C; ^14^ FF × S, interaction between formulation factor and storage time. Different superscript letters within rows indicate significant differences; lowercase letters indicate significant differences between levels of the formulation factor (FF); uppercase letters indicate significant differences among levels of the storage time factor (S).

**Table 4:** Sugar content of *Casu Axedu* enriched with inulin and olive leaf extract and inoculated with *Lactiplantibacillus plantarum* UNICA B26 during storage.

	Formulation Factor			Storage Time at 3 ± 1 °C				*p*-Value ^5^		
	CA-I ^1^	CA-IL ^2^	CA-IOL ^3^	1 Day	15 Days	30 Days	SEM ^4^	FF ^6^	S ^7^	FF × S ^8^
Total sugars (%)	2.0	2.2	2.3	2.5	2.1	1.9	0.4	ns	ns	ns
(γ + α + β) galactose (%)	1.2	1.3	1.4	1.5	1.2	1.2	0.2	ns	ns	ns
(α + β) glucose (%)	0.7	0.8	0.9	0.9	0.8	0.7	0.1	ns	ns	ns
(α + β) lactose (%)	0.007	0.006	0.009	0.011	0.005	0.005	0.004	ns	ns	ns
myo-inositol (%)	0.014	0.014	0.017	0.018	0.015	0.012	0.003	ns	ns	ns
(α + β) tagatose (%)	0.004	0.005	0.006	0.005	0.005	0.005	0.001	ns	ns	ns

^1^ CA-I, *Casu Axedu* cheese fortified with inulin (DP ≥ 20) (control); ^2^ CA-IL, *Casu Axedu* cheese fortified with inulin (DP ≥ 20) and *Lactiplantibacillus plantarum* (UNICA B26); ^3^ CA-IOL, *Casu Axedu* cheese fortified with inulin (DP ≥ 20) and OLE (TP = 20 mg GAE 100 g^−1^ of milk) and *Lactiplantibacillus plantarum* (UNICA B26); ^4^ SEM, standard error of the mean; ^5^ *p*-value, ns, no significant difference (*p* > 0.05); ^6^ FF, formulation factor; ^7^ S, storage time factor at 3 ± 1 °C; ^8^ FF × S, interaction between formulation factor and storage time.

**Table 5:** Fatty acid profile, vitamins, and cholesterol content of *Casu Axedu* enriched with inulin and olive leaf extract and inoculated with *Lactiplantibacillus plantarum* UNICA B26 during storage.

	Formulation Factor			Storage Time at 3 ± 1 °C				*p*-Value ^14^		
	CA-I ^1^	CA-IL ^2^	CA-IOL ^3^	1 Day	15 Days	30 Days	SEM ^13^	FF ^15^	S ^16^	FF × S ^17^
Fatty acid profile expressed as % of FAME										
SFA ^4^	69.9	69.9	69.9	69.9	69.9	69.9	0.3	ns	ns	ns
UFA ^5^	30.1	30.1	30.1	30.1	30.1	30.1	0.3	ns	ns	ns
TTFA ^6^	3.1	3.1	3.1	3.1	3.1	3.1	0.1	ns	ns	ns
MUFA ^7^	21.9	21.8	21.8	21.8	21.8	21.9	0.4	ns	ns	ns
PUFA ^8^	3.8	3.8	3.8	3.8	3.9	3.8	0.2	ns	ns	ns
CLA cis9 trans 11 ^9^	0.76	0.75	0.76	0.76	0.76	0.76	0.03	ns	ns	ns
PUFA n-6 ^10^	2.7	2.7	2.7	2.7	2.7	2.7	0.1	ns	ns	ns
PUFA n-3 ^11^	1.1	1.1	1.1	1.1	1.2	1.1	0.1	ns	ns	ns
PUFA n-6/PUFA n-3 ^12^	2.4	2.4	2.4	2.4	2.4	2.4	0.2	ns	ns	ns
Vitamins and cholesterol										
Vitamin A (ppm)	0.96 ^a^	0.93 ^a^	0.84 ^b^	0.93	0.88	0.91	0.04	*	ns	ns
Vitamin E (ppm)	3.3	3.3	3.1	3.3	3.1	3.2	0.4	ns	ns	ns
Cholesterol (ppm)	313 ^a^	299 ^ab^	290 ^b^	306	298	298	10	*	ns	ns

^1^ CA-I, *Casu Axedu* cheese fortified with inulin (DP ≥ 20) (control); ^2^ CA-IL, *Casu Axedu* cheese fortified with inulin (DP ≥ 20) and *Lactiplantibacillus plantarum* (UNICA B26); ^3^ CA-IOL, *Casu Axedu* cheese fortified with inulin (DP ≥ 20) and OLE (TP = 20 mg GAE 100 g^−1^ of milk) and *Lactiplantibacillus plantarum* (UNICA B26); ^4^ SFA, saturated fatty acids; ^5^ UFA, unsaturated fatty acids; ^6^ TTFA, total trans fatty acids; ^7^ MUFA, monounsaturated fatty acids (only cis isomers, as per Reg. (EU) No 1169/2011); ^8^ PUFA, polyunsaturated fatty acids; ^9^ CLA cis9 trans11–conjugated linoleic acid (cis-9, trans-11 isomer); ^10^ PUFA n-6, omega-6 polyunsaturated fatty acids; ^11^ PUFA n-3, omega-3 polyunsaturated fatty acids; ^12^ PUFA n-6/PUFA n-3, ratio between omega-6 and omega-3 polyunsaturated fatty acids; ^13^ SEM, standard error of the mean; ^14^ *p*-value, ns, no significant difference (*p* > 0.05); * *p* < 0.05; ^15^ FF, formulation factor; ^16^ S, storage time factor at 3 ± 1 °C; ^17^ FF × S, interaction between formulation factor and storage time. Different superscript letters within rows indicate significant differences; lowercase letters indicate significant differences between levels of the formulation factor (FF).

**Table 6:** Texture, water holding capacity, and colorimetric parameters of *Casu Axedu* enriched with inulin and olive leaf extract and inoculated with *Lactiplantibacillus plantarum* UNICA B26 during storage.

	Formulation Factor			Storage Time at 3 ± 1 °C				*p*-Value ^7^		
	CA-I ^1^	CA-IL ^2^	CA-IOL ^3^	1 Day	15 Days	30 Days	SEM ^6^	FF ^8^	S ^9^	FF × S ^10^
Penetrometry parameters										
*F*_max_ (N)	2.2 ^a^	2.1 ^a^	1.6 ^b^	2.2 ^A^	2.0 ^AB^	1.7 ^B^	0.2	*	*	ns
Positive area (N mm)	26	25	23	26	26	23	2	ns	ns	ns
Negative *F*_max_ (N)	−0.57 ^b^	−0.53 ^ab^	−0.41 ^a^	−0.46	−0.54	−0.50	0.06	*	ns	ns
Negative area (N mm)	−6.4 ^b^	−5.9 ^b^	−4.4 ^a^	−5.0 ^A^	−6.1 ^B^	−5.6 ^AB^	0.4	*	*	ns
Whey released										
Syneresis (%) ^4^	30 ^a^	30 ^a^	23 ^b^	28	27	27	3	*	ns	ns
Colorimetric parameters										
L*	89	89	88	89	88	89	1	*	ns	ns
a*	−2.8 ^b^	−2.6 ^b^	−2.2 ^a^	−2.5	−2.4	−2.6	0.2	*	ns	ns
b*	8.1 ^b^	7.8 ^ab^	8.7 ^a^	8.2	8.0	8.4	0.3	*	ns	ns
ΔE ^5^ (distance from CA-I vs. CA-IL)		0.6		0.6	0.6	0.6	0.1	-	ns	-
ΔE ^5^ (distance from CA-I vs. CA-IOL)		1.5		1.7	1.3	1.5	0.2	-	ns	-
ΔE ^5^ (distance from CA-IL vs. CA-IOL)		1.6		1.7	1.4	1.7	0.1	-	ns	-

^1^ CA-I, *Casu Axedu* cheese fortified with inulin (DP ≥ 20) (control); ^2^ CA-IL, *Casu Axedu* cheese fortified with inulin (DP ≥ 20) and *Lactiplantibacillus plantarum* (UNICA B26); ^3^ CA-IOL, *Casu Axedu* cheese fortified with inulin (DP ≥ 20) and OLE (TP = 20 mg GAE 100 g^−1^ of milk) and *Lactiplantibacillus plantarum* (UNICA B26); ^4^ Syneresis was expressed as the percentage of whey released from the *Casu Axedu* cheese, calculated on 100 g of total product (cheese + whey); ^5^ ΔE represents the colour difference between samples in the CIELAB colour space and is calculated as: ΔE = √[(*L*_2_ *− L*_1_)^2^ + (*a*_2_ − *a*_1_)^2^ + (*b*_2_ − *b*_1_)^2^]; ^6^ SEM, standard error of the mean; ^7^ *p*-value, ns, no significant difference (*p* > 0.05); * *p* < 0.05; ^8^ FF, formulation factor; ^9^ S, storage time factor at 3 ± 1 °C; ^10^ FF × S, interaction between formulation factor and storage time. Different superscript letters within rows indicate significant differences; lowercase letters indicate significant differences between levels of the formulation factor (FF); uppercase letters indicate significant differences among levels of the storage time factor (S).

**Table 7:** Mean intensity scores of the sensory descriptors obtained by quantitative descriptive analysis (QDA) for the three experimental formulations evaluated at the three shelf-life times. *p*-values from two-way ANOVA for formulation (FF) and shelf-life (S) effects are also reported. QDA data were analysed by ANOVA, including formulation factor (FF), storage time (S), and assessor; assessor was included in the model as a random factor to account for panel variability. For clarity, only the *p*-values for FF and S are reported, as the assessor effect was not significant (*p* > 0.05). Different lowercase and uppercase letters, when present, indicate significant differences (*p* ≤ 0.05) among mean liking scores for formulation and storage time, respectively. ns, no significant difference (*p* > 0.05).

	Formulation Factor			Storage Time at 3 ± 1 °C			*p*-Value	
	CA-I ^1^	CA-IL ^2^	CA-IOL ^3^	1 Day	15 Days	30 Days	FF	S
Colour intensity	2.71 ^b^ ± 0.87	2.83 ^b^ ± 0.86	3.31 ^a^ ± 0.92	3.14 ^AB^ ± 0.87	2.72 ^B^ ± 1.01	3.04 ^A^ ± 0.81	<0.001	0.03
Acidic odour	4.23 ± 0.77	4.27 ± 0.72	4.38 ± 0.91	4.32 ± 0.73	4.13 ± 0.80	4.45 ± 0.83	ns	ns
Sour taste	4.43 ^b^ ± 1.29	4.83 ^a^ ± 1.18	4.77 ^a^ ± 1.53	4.85 ^B^ ± 0.93	4.42 ^AB^ ± 1.55	4.81 ^A^ ± 1.37	0.020	0.030
Sweet taste	3.52 ^a^ ± 1.00	3.35 ^ab^ ± 1.04	3.24 ^b^ ± 1.08	3.36 ± 0.95	3.46 ± 1.07	3.29 ± 1.09	0.020	ns
Bitter taste	2.42 ^b^ ± 1.21	2.41 ^b^ ± 1.13	3.57 ^a^ ± 1.59	2.59 ^B^ ± 1.48	2.73 ^B^ ± 1.53	3.05 ^A^ ± 1.25	<0.001	<0.001
Acidic aroma	4.32 ± 0.84	4.48 ± 0.91	4.45 ± 0.96	4.44 ± 0.79	4.21 ± 0.87	4.63 ± 0.98	ns	ns
Fresh milk aroma	3.58 ^a^ ± 1.16	3.62 ^a^ ± 1.22	3.29 ^b^ ± 1.10	3.38 ± 1.16	3.44 ± 1.12	3.65 ± 1.21	<0.001	ns
Sheep milk aroma	4.23 ± 0.80	4.23 ± 0.84	4.11 ± 0.67	4.15 ± 0.77	4.06 ± 0.70	4.37 ± 0.82	ns	ns
Olive oil aroma	2.18 ^b^ ± 0.97	2.24 ^b^ ± 0.97	3.30 ^a^ ± 1.30	2.51 ± 1.16	2.65 ± 1.28	2.54 ± 1.15	<0.001	ns
Cohesiveness	4.32 ± 1.19	3.99 ± 1.15	4.14 ± 1.25	4.27 ± 1.18	4.10 ± 1.22	4.11 ± 1.20	ns	ns
Astringency	3.59 ^b^ ± 1.01	3.75 ^b^ ± 1.24	4.16 ^a^ ± 1.12	3.72 ± 1.09	3.91 ± 1.13	3.85 ± 1.22	<0.001	ns
Watery consistency	3.56 ± 1.81	3.79 ± 1.66	3.81 ± 1.71	3.81 ± 1.89	3.80 ± 1.70	3.56 ± 1.60	ns	ns
Off-flavour	1.57 ^b^ ± 0.83	1.82 ^b^ ± 1.25	2.57 ^a^ ± 2.02	1.81 ± 1.25	1.87 ± 1.62	2.27 ± 1.56	<0.001	ns
Overall sensory quality	6.03 ^a^ ± 0.69	5.77 ^a^ ± 1.13	4.84 ^b^ ± 1.30	5.87 ^A^ ± 0.91	5.60 ^AB^ ± 1.21	5.22 ^B^ ± 1.28	<0.001	0.020

^1^ CA-I, *Casu Axedu* cheese fortified with inulin (DP ≥ 20) (control); ^2^ CA-IL, *Casu Axedu* cheese fortified with inulin (DP ≥ 20) and *Lactiplantibacillus plantarum* (UNICA B26); ^3^ CA-IOL, *Casu Axedu* cheese fortified with inulin (DP ≥ 20) and OLE (TP = 20 mg GAE 100 g^−1^ of milk) and *Lactiplantibacillus plantarum* (UNICA B26).

**Table 8:** Consumer test results for the experimental cheeses evaluated at 1 and 30 days of storage, including overall liking scores on a 9-point hedonic scale and citation frequencies for the CATA terms. Overall liking data, including formulation and consumer at each storage time, were analysed by ANOVA. The consumer effect was significant (*p* ≤ 0.05), reflecting inter-individual variability in liking responses. For clarity, only formulation-related comparisons at each storage time are reported in the table. *p*-values from ANOVA are reported for overall liking, whereas *p*-values from Cochran’s Q test are reported for each CATA term at each storage time. Different letters, when present, indicate significant differences among mean liking scores (*p* ≤ 0.05).

	1 Day Storage				30 Days Storage			
	CA-I ^1^	CA-IL ^2^	CA-IOL ^3^	*p*-Value	CA-I ^1^	CA-IL ^2^	CA-IOL ^3^	*p*-Value
Liking	6.33 ^a^ ± 1.6	6.13 ^ab^ ± 1.5	5.61 ^b^ ± 1.5	0.0237	6.1 ^a^ ± 1.6	6.49 ^a^ ± 1.6	5.26 ^b^ ±1.7	<0.001
Sour taste	0.293	0.268	0.463	0.058	0.548	0.516	0.419	0.444
Sweet taste	0.244 ^a^	0.098 ^ab^	0.049 ^b^	0.018	0.097 ^a^	0.129 ^a^	0.032 ^a^	0.311
Bitter taste	0.049 ^b^	0.049 ^b^	0.366 ^a^	<0.0001	0.129 ^b^	0.097 ^b^	0.645 ^a^	<0.0001
Fresh milk aroma	0.415 ^a^	0.268 ^ab^	0.122 ^b^	0.003	0.290	0.226	0.129	0.178
Sheep milk aroma	0.195	0.220	0.146	0.607	0.194	0.258	0.097	0.150
Buttery aroma	0.049	0.146	0.073	0.074	0.161	0.194	0.065	0.307
Fresh grass aroma (reminiscent of thistle/artichoke)	0.073	0.146	0.122	0.497	0.065	0.129	0.194	0.336
Olive oil aroma	0.073	0.049	0.146	0.236	0.097 ^b^	0.065 ^b^	0.258 ^a^	0.006
Astringent mouthfeel	0.146	0.195	0.268	0.257	0.258	0.290	0.323	0.819
Cohesive texture (pudding-like consistency)	0.244	0.268	0.146	0.174	0.226	0.226	0.194	0.913
Presence of lumps	0.098	0.220	0.220	0.125	0.032	0.129	0.032	0.050
Watery texture	0.195	0.146	0.122	0.417	0.194	0.226	0.097	0.115
Traditional	0.195	0.146	0.073	0.232	0.226 ^a^	0.194 ^ab^	0.032 ^b^	0.032
Presence of off-flavours/off-odours	0.049	0.049	0.098	0.565	0.129	0	0.032	0.074
Balanced taste	0.317	0.293	0.220	0.522	0.452 ^a^	0.419 ^a^	0.097 ^b^	0.001
Rich taste	0.220	0.098	0.073	0.076	0.065	0.194	0.129	0.264
Flat taste	0.098	0.098	0.098	1.000	0.065	0.065	0.000	0.368
Cloying/tiring on the palate	0.024	0.049	0.098	0.247	0.032	0	0.129	0.074
Unpleasant/annoying	0.049	0	0.098	0.135	0.161	0.097	0.290	0.078
Natural	0.366	0.488	0.293	0.108	0.419 ^a^	0.419 ^a^	0.194 ^b^	0.047
Healthy	0.122	0.098	0.098	0.846	0.129	0.129	0.129	1.000
Smooth/soft texture	0.488	0.585	0.390	0.091	0.290	0.290	0.323	0.920

^1^ CA-I, *Casu Axedu* cheese fortified with inulin (DP ≥ 20) (control); ^2^ CA-IL, *Casu Axedu* cheese fortified with inulin (DP ≥ 20) and *Lactiplantibacillus plantarum* (UNICA B26); ^3^ CA-IOL, *Casu Axedu* cheese fortified with inulin (DP ≥ 20) and OLE (TP = 20 mg GAE 100 g^−1^ of milk) and *Lactiplantibacillus plantarum* (UNICA B26).

## Data Availability

The original contributions presented in the study are included in the article/Supplementary Materials; further inquiries can be directed to the corresponding author.

## Supplementary Materials

The following supporting information can be downloaded at: https://www.mdpi.com/article/10.3390/foods15152694/s1, Figure S1: Flow diagram of the pilot-scale production of *Casu Axedu* enriched with inulin and olive leaf extract and inoculated with *Lactiplantibacillus plantarum* strain; Figure S2. Chromatogram at λ = 280 nm of OLE (diluted 1:200 *v*/*v* with 0.22 M H_3_PO_4_) L-glu: luteolin-7-*O*-glucoside, Verb: verbascoside, HTyr: hydroxytyrosol, HTyr-glu: hydroxytyrosol glucoside, Olup: oleuropein; Figure S3: Evolution of target phenolic compounds in fortified *Casu Axedu* L-glu: luteolin-7-*O*-glucoside, Verb: verbascoside, HTyr: hydroxytyrosol, HTyr-glu: hydroxytyrosol glucoside, Olup: oleuropein; Figure S4: Penetration profile of *Casu Axedu* cheese (CA-I, *Casu Axedu* cheese fortified with inulin (DP ≥ 20) (control); CA-IL, *Casu Axedu* cheese fortified with inulin (DP ≥ 20) and *Lactiplantibacillus plantarum* UNICA B26; CA-IOL, *Casu Axedu* cheese fortified with inulin (DP ≥ 20), OLE (TP = 20 mg GAE 100 g^−1^ of milk) and *Lactiplantibacillus plantarum* UNICA B26; Figure S5: Penetration profile of *Casu Axedu* cheese during storage time at 3 ± 1 °C; Figure S6: Multiple factor analysis (MFA) of QDA, CATA, and hedonic data collected at the storage times common to all datasets. (A) Individual factor map of the experimental cheeses. (B) Variable factor map. Blue labels correspond to QDA attributes, red labels to CATA terms, and green labels to hedonic variables (trained-panel overall sensory quality and consumer overall liking). For readability, “aroma” was abbreviated as “Ar” and “milk” as “m” in the variable labels. Variables projected in the same area are positively associated, whereas variables located on opposite sides are negatively associated. The MFA highlights the opposition between bitter taste, astringency/astringent mouthfeel, and olive oil aroma, and the positive hedonic descriptors associated with liking, overall sensory quality, balanced taste, naturalness, and traditionality; Table S1: Analytical parameters of HPLC-PDA quantitative method. Data for calibration curves (calibration range and linearity), limit of detection (LOD), limit of quantification (LOQ) and recovery (%) values are reported for each target phenolic compound dosed by pure standard; Table S2: List of attributes used for quantitative descriptive analysis. For each attribute, the sensory definition, reference standards, and corresponding intensity values (in parentheses) employed for judge calibration are provided; Table S3: Free fatty acids content and proteolysis indices of *Casu Axedu* enriched with inulin and olive leaf extract and inoculated with *Lactiplantibacillus plantarum* UNICA B26 during storage; Table S4. Acidification kinetic parameters of *Casu Axedu* enriched with inulin and olive leaf extract and inoculated with *Lactiplantibacillus plantarum* UNICA B26 during fermentation; Table S5: RV coefficients among QDA, CATA and EDO datasets and the global MFA configuration.

## References

[1] Martins N., Oliveira M.B.P.P., Ferreira I.C.F.R., Mérillon J.-M., Ramawat K.G. (2017). Development of Functional Dairy Foods. Bioactive Molecules in Food.

[2] Fekete M., Lehoczki A., Kryczyk-Poprawa A., Zábó V., Varga J.T., Bálint M., Fazekas-Pongor V., Csípő T., Rząsa-Duran E., Varga P. (2025). Functional Foods in Modern Nutrition Science: Mechanisms, Evidence, and Public Health Implications. Nutrients.

[3] Wazzan H. (2024). Fortification of Dairy Products Using Plant-Derived Bioactive Compounds. Curr. Res. Nutr. Food Sci..

[4] Rolim F.R.L., Freitas Neto O.C., Oliveira M.E.G., Oliveira C.J.B., Queiroga R.C.R.E. (2020). Cheeses as Food Matrixes for Probiotics: In Vitro and in Vivo Tests. Trends Food Sci. Technol..

[5] Milani C., Longhi G., Alessandri G., Fontana F., Viglioli M., Tarracchini C., Mancabelli L., Lugli G.A., Petraro S., Argentini C. (2025). Functional Modulation of the Human Gut Microbiome by Bacteria Vehicled by Cheese. Appl. Environ. Microbiol..

[6] De Oliveira C.M.S., Grisi C.V.B., Silva G.D.S., Lopes Neto J.H.P., De Medeiros L.L., Dos Santos K.M.O., Cardarelli H.R. (2023). Use of Lactiplantibacillus Plantarum CNPC 003 for the Manufacture of Functional Skimmed Fresh Cheese. Int. Dairy J..

[7] Chugh B., Kamal-Eldin A. (2020). Bioactive Compounds Produced by Probiotics in Food Products. Curr. Opin. Food Sci..

[8] Zhang Z., Niu H., Qu Q., Guo D., Wan X., Yang Q., Mo Z., Tan S., Xiang Q., Tian X. (2025). Advancements in Lactiplantibacillus Plantarum: Probiotic Characteristics, Gene Editing Technologies and Applications. Crit. Rev. Food Sci. Nutr..

[9] Ngamsomchat A., Kaewkod T., Konkit M., Tragoolpua Y., Bovonsombut S., Chitov T. (2022). Characterisation of Lactobacillus Plantarum of Dairy-Product Origin for Probiotic Chèvre Cheese Production. Foods.

[10] Arshad Z., Shahid S., Hasnain A., Yaseen E., Rahimi M. (2025). Functional Foods Enriched With Bioactive Compounds: Therapeutic Potential and Technological Innovations. Food Sci. Nutr..

[11] Šimat V., Skroza D., Tabanelli G., Čagalj M., Pasini F., Gómez-Caravaca A.M., Fernández-Fernández C., Sterniša M., Smole Možina S., Ozogul Y. (2022). Antioxidant and Antimicrobial Activity of Hydroethanolic Leaf Extracts from Six Mediterranean Olive Cultivars. Antioxidants.

[12] Zoidou E., Magiatis P., Melliou E., Constantinou M., Haroutounian S., Skaltsounis A.-L. (2014). Oleuropein as a Bioactive Constituent Added in Milk and Yogurt. Food Chem..

[13] Regolo L., Giampieri F., Battino M., Armas Diaz Y., Mezzetti B., Elexpuru-Zabaleta M., Mazas C., Tutusaus K., Mazzoni L. (2024). From by-Products to New Application Opportunities: The Enhancement of the Leaves Deriving from the Fruit Plants for New Potential Healthy Products. Front. Nutr..

[14] Tarchi I., Bouaziz M., Bhat Z.F., Aït-Kaddour A. (2024). Effect of Olive Leaf Extract on the Quality of Cantal Cheese. Food Chem. X.

[15] Barukčić I., Filipan K., Lisak Jakopović K., Božanić R., Blažić M., Repajić M. (2022). The Potential of Olive Leaf Extract as a Functional Ingredient in Yoghurt Production: The Effects on Fermentation, Rheology, Sensory, and Antioxidant Properties of Cow Milk Yoghurt. Foods.

[16] Caponio F., Difonzo G., Calasso M., Cosmai L., De Angelis M. (2019). Effects of Olive Leaf Extract Addition on Fermentative and Oxidative Processes of Table Olives and Their Nutritional Properties. Food Res. Int..

[17] Selim S., Albqmi M., Al-Sanea M.M., Alnusaire T.S., Almuhayawi M.S., AbdElgawad H., Al Jaouni S.K., Elkelish A., Hussein S., Warrad M. (2022). Valorizing the usage of olive leaves, bioactive compounds, biological activities, and food applications: A comprehensive review. Front. Nutr..

[18] Hughes R.L., Alvarado D.A., Swanson K.S., Holscher H.D. (2022). The Prebiotic Potential of Inulin-Type Fructans: A Systematic Review. Adv. Nutr..

[19] Perinelli D.R., Santanatoglia A., Caprioli G., Bonacucina G., Vittori S., Maggi F., Sagratini G. (2023). Inulin Functionalized “Giuncata” Cheese as a Source of Prebiotic Fibers. Foods.

[20] Li A., Zheng J., Han X., Yang S., Cheng S., Zhao J., Zhou W., Lu Y. (2023). Advances in Low-Lactose/Lactose-Free Dairy Products and Their Production. Foods.

[21] (2025). List of Traditional Italian Agri-Food Products, Twenty-Fifth Revision. https://www.masaf.gov.it/flex/cm/pages/ServeBLOB.php/L/IT/IDPagina/22781.

[22] Spanu C., Scarano C., Venusti M., Sardo D., Serra S., Ibba M., Frau F., De Santis E.P.L. (2013). Hygienic and Sensory Quality Factors Affecting the Shelf-Life of Fruhe (*Casu Axedu*) Traditional Sardinian Fresh Cheese. Ital. J. Food Saf..

[23] Abbondio M., Palomba A., Serra M., Fraumene C., Di Meo C., Marongiu F., Sau R., Pagnozzi D., Laconi E., Tanca A. (2025). Consumption of Traditional Sardinian Fermented Milk Promotes Changes in the Rat Gut Microbiota Composition and Functions. BMC Microbiol..

[24] Gil K.A., Jerković I., Marijanović Z., Manca M.L., Caddeo C., Tuberoso C.I.G. (2022). Evaluation of an Innovative Sheep Cheese with Antioxidant Activity Enriched with Different Thyme Essential Oil Lecithin Liposomes. LWT.

[25] Pisano M.B., Viale S., Conti S., Fadda M.E., Deplano M., Melis M.P., Deiana M., Cosentino S. (2014). Preliminary Evaluation of Probiotic Properties of Lactobacillus Strains Isolated from Sardinian Dairy Products. BioMed. Res. Int..

[26] Pisano M.B., Rosa A., Putzu D., Cesare Marincola F., Mossa V., Viale S., Fadda M.E., Cosentino S. (2020). Influence of Autochthonous Putative Probiotic Cultures on Microbiota, Lipid Components and Metabolome of Caciotta Cheese. Front. Microbiol..

[27] Rychen G., Aquilina G., Azimonti G., Bampidis V., de Lourdes Bastos M., Bories G., Chesson A., Cocconcelli P.S., Flachowsky G., EFSA Panel on Additives and Products or Substances Used in Animal Feed (FEEDAP) (2018). Guidance on the Characterisation of Microorganisms Used as Feed Additives or as Production Organisms. EFSA J..

[28] Spinnler H.E., Corrieu G. (1989). Automatic Method to Quantify Starter Activity Based on pH Measurement. J. Dairy Res..

[29] De Luca M., Lucchesi D., Tuberoso C.I.G., Fernàndez-Busquets X., Vassallo A., Martelli G., Fadda A.M., Pucci L., Caddeo C. (2022). Liposomal Formulations to Improve Antioxidant Power of Myrtle Berry Extract for Potential Skin Application. Pharmaceutics.

[30] Abbattista R., Ventura G., Calvano C.D., Cataldi T.R.I., Losito I. (2021). Bioactive Compounds in Waste By-Products from Olive Oil Production: Applications and Structural Characterization by Mass Spectrometry Techniques. Foods.

[31] Fu S., Arráez-Roman D., Segura-Carretero A., Menéndez J.A., Menéndez-Gutiérrez M.P., Micol V., Fernández-Gutiérrez A. (2010). Qualitative Screening of Phenolic Compounds in Olive Leaf Extracts by Hyphenated Liquid Chromatography and Preliminary Evaluation of Cytotoxic Activity against Human Breast Cancer Cells. Anal. Bioanal. Chem..

[32] Kabbash E.M., Abdel-Shakour Z.T., El-Ahmady S.H., Wink M., Ayoub I.M. (2023). Comparative Metabolic Profiling of Olive Leaf Extracts from Twelve Different Cultivars Collected in Both Fruiting and Flowering Seasons. Sci. Rep..

[33] Kontogianni V.G., Charisiadis P., Margianni E., Lamari F.N., Gerothanassis I.P., Tzakos A.G. (2013). Olive Leaf Extracts Are a Natural Source of Advanced Glycation End Product Inhibitors. J. Med. Food.

[34] Olmo-García L., Bajoub A., Benlamaalam S., Hurtado-Fernández E., Bagur-González M.G., Chigr M., Mbarki M., Fernández-Gutiérrez A., Carrasco-Pancorbo A. (2018). Establishing the Phenolic Composition of *Olea europaea* L. Leaves from Cultivars Grown in Morocco as a Crucial Step Towards Their Subsequent Exploitation. Molecules.

[35] Romani A., Scardigli A., Pinelli P. (2017). An Environmentally Friendly Process for the Production of Extracts Rich in Phenolic Antioxidants from *Olea europaea* L. and Cynara *Scolymus* L. Matrices. Eur. Food Res. Technol..

[36] Romero-Márquez J.M., Navarro-Hortal M.D., Forbes-Hernández T.Y., Varela-López A., Puentes J.G., Sánchez-González C., Sumalla-Cano S., Battino M., García-Ruiz R., Sánchez S. (2024). Effect of Olive Leaf Phytochemicals on the Anti-Acetylcholinesterase, Anti-Cyclooxygenase-2 and Ferric Reducing Antioxidant Capacity. Food Chem..

[37] (2010). Milk, Cream and Evaporated Milk—Determination of Total Solids Content (Reference Method).

[38] Vázquez C.V., Rojas M.G.V., Ramírez C.A., Chávez-Servín J.L., García-Gasca T., Ferriz Martínez R.A., García O.P., Rosado J.L., López-Sabater C.M., Castellote A.I. (2015). Total Phenolic Compounds in Milk from Different Species. Design of an Extraction Technique for Quantification Using the Folin–Ciocalteu Method. Food Chem..

[39] Abd El-Fattah A., Azzam M., Elkashef H., Elhadydy A. (2019). Antioxidant Properties of Milk: Effect of Milk Species, Milk Fractions and Heat Treatments. Int. J. Dairy Sci..

[40] Benzie I.F.F., Strain J.J. (1996). The Ferric Reducing Ability of Plasma (FRAP) as a Measure of “Antioxidant Power”: The FRAP Assay. Anal. Biochem..

[41] (2004). Cheese and Processed Cheese—Determination of the Total Solids Content.

[42] Soxhlet F. (1879). Die gewichtsanalytische Bestimmung des Milchfettes. Dingler’s Polyt. J..

[43] (2014). Milk and Milk Products—Determination of Nitrogen Content—Part 1: Kjeldahl Principle and Crude Protein Calculation.

[44] Yilmaz-Ersan L., Ozcan T., Akpinar-Bayizit A., Sahin S. (2018). Comparison of Antioxidant Capacity of Cow and Ewe Milk Kefirs. J. Dairy Sci..

[45] Dedola A.S., Caredda M., Addis M., Lai G., Fiori M., Pes M., Mara A., Sanna G. (2024). Determining Carbohydrates for Increasing Safety: GC-FID Quantification of Lactose, Galactose, Glucose, Tagatose and Myo-Inositol in ‘Maturo’ PDO Pecorino Sardo Cheese. Separations.

[46] Addis M., Fiori M., Riu G., Pes M., Salvatore E., Pirisi A. (2015). Physico-Chemical Characteristics and Acidic Profile of PDO Pecorino Romano Cheese: Seasonal Variation. Small Rumin. Res..

[47] Panfili G., Manzi P., Pizzoferrato L. (1994). High-Performance Liquid Chromatographic Method for the Simultaneous Determination of Tocopherols, Carotenes, and Retinol and Its Geometric Isomers in Italian Cheeses. Analyst.

[48] Manzi P., Panfili G., Pizzoferrato L. (1996). Normal and Reversed-Phase HPLC for More Complete Evaluation of Tocopherols, Retinols, Carotenes and Sterols in Dairy Products. Chromatographia.

[49] Addis M., Piredda G., Pes M., Di Salvo R., Scintu M.F., Pirisi A. (2005). Effect of the Use of Three Different Lamb Paste Rennets on Lipolysis of the PDO Pecorino Romano Cheese. Int. Dairy J..

[50] Gripon J.C., Desmazeaud M.J., Le Bars D., Bergere J.L. (1975). Etude Du Rôle Des Micro-Organismes et Des Enzymes Au Cours de La Maturation Des Fromages. II.—Influence de La Présure Commerciale. Lait.

[51] European Parliament and Council of the European Union Regulation (EC) No 1924/2006 of the European Parliament and of the Council of 20 December 2006 on Nutrition and Health Claims Made on Foods (OJ L 404, 30.12.2006, pp. 9–25). EUR-Lex. 2006. https://eur-lex.europa.eu/legal-content/EN/TXT/?uri=CELEX:32006R1924.

[52] European Parliament and Council of the European Union Regulation (EU) No 1169/2011 of the European Parliament and of the Council of 25 October 2011 on the Provision of Food Information to Consumers (OJ L 304, 22.11.2011, pp. 18–63). EUR-Lex. 2011. https://eur-lex.europa.eu/legal-content/EN/TXT/?uri=CELEX:32011R1169.

[53] Salvatore E., Pes M., Mazzarello V., Pirisi A. (2014). Replacement of Fat with Long-Chain Inulin in a Fresh Cheese Made from Caprine Milk. Int. Dairy J..

[54] Aryana K.J., McGrew P. (2007). Quality Attributes of Yogurt with Lactobacillus Casei and Various Prebiotics. LWT-Food Sci. Technol..

[55] Lawless H.T., Heymann H., Lawless H.T., Heymann H. (2010). Descriptive Analysis. Sensory Evaluation of Food: Principles and Practices.

[56] (2023). Sensory Analysis—Selection and Training of Sensory Assessors.

[57] Lawless H.T., Heymann H., Lawless H.T., Heymann H. (2010). Acceptance Testing. Sensory Evaluation of Food: Principles and Practices.

[58] Ares G., Jaeger S.R., Delarue J., Lawlor J.B., Rogeaux M. (2015). 11—Check-All-That-Apply (CATA) Questions with Consumers in Practice: Experimental Considerations and Impact on Outcome. Rapid Sensory Profiling Techniques.

[59] (2014). Sensory Analysis—Methodology—General Guidance for Conducting Hedonic Tests with Consumers in a Controlled Area.

[60] MacFie H.J., Bratchell N., Greenhoff K., Vallis L.V. (1989). Designs to Balance the Effect Of Order Of Presentation and First-Order Carry-Over Effects in Hall Tests. J. Sens. Stud..

[61] (2007). Sensory Analysis—General Guidance for the Design of Test Rooms.

[62] (2016). Sensory Analysis—Methodology—General Guidance for Establishing a Sensory Profile.

[63] (2023). Milk and Milk Products—Sensory Analysis—Part 2: Methods for Sensory Evaluation.

[64] Institute of Food Science and Technology (IFST) (2020). Guidelines for Ethical and Professional Practices for the Sensory Analysis of Foods. https://www.ifst.org.

[65] Næs T., Brockhoff P.B., Tomic O. (2010). Statistics for Sensory and Consumer Science.

[66] Hervert D., Goñi I. (2011). Dietary Polyphenols and Human Gut Microbiota: A Review. Food Rev. Int..

[67] Iorizzo M., Lombardi S.J., Macciola V., Testa B., Lustrato G., Lopez F., De Leonardis A. (2016). Technological Potential of Lactobacillus Strains Isolated from Fermented Green Olives: In Vitro Studies with Emphasis on Oleuropein-Degrading Capability. Sci. World J..

[68] Landete J.M., Curiel J.A., Rodríguez H., Rivas B.d.l., Muñoz R. (2008). Study of the Inhibitory Activity of Phenolic Compounds Found in Olive Products and Their Degradation by Lactobacillus Plantarum Strains. Food Chem..

[69] Zhang K., Huang J., Wang D., Wan X., Wang Y. (2024). Covalent Polyphenols-Proteins Interactions in Food Processing: Formation Mechanisms, Quantification Methods, Bioactive Effects, and Applications. Front. Nutr..

[70] Wróblewska B., Kuliga A., Wnorowska K. (2023). Bioactive Dairy-Fermented Products and Phenolic Compounds: Together or Apart. Molecules.

[71] Farkas Z., Romeo R., Pangallo D., Kraková L., Giuffrè A.M., Sidari R. (2025). Conversion of oleuropein to hydroxytyrosol by lactic acid bacteria fermentation of olive leaves in water solution with reduced glucose content. World J. Microbiol. Biotechnol..

[72] Carboni A., Cabizza R., Urgeghe P.P., Fancello F., Zara S., Del Caro A. (2025). Effects of Olive Pomace Powder Incorporation on Physicochemical, Textural, and Rheological Properties of Sheep Milk Yogurt. Foods.

[73] Sik B., Székelyhidi R., Lakatos E., Kapcsándi V., Ajtony Z. (2022). Analytical Procedures for Determination of Phenolics Active Herbal Ingredients in Fortified Functional Foods: An Overview. Eur. Food Res. Technol..

[74] Clodoveo M.L., Crupi P., Annunziato A., Corbo F. (2022). Innovative Extraction Technologies for Development of Functional Ingredients Based on Polyphenols from Olive Leaves. Foods.

[75] Romani A., Campo M., Urciuoli S., Marrone G., Noce A., Bernini R. (2020). An Industrial and Sustainable Platform for the Production of Bioactive Micronized Powders and Extracts Enriched in Polyphenols from *Olea Europaea* L. and *Vitis Vinifera* L. Wastes. Front. Nutr..

[76] Zandona E., Vukelić M., Hanousek Čiča K., Zandona A., Mrvčić J., Katalinić M., Cindrić I., Abdurramani A., Jurina I.B. (2025). Bioactive Properties of the Microwave-Assisted Olive Leaf Extract and Its Incorporation into a Whey Protein Isolate Coating of Semi-Hard Cheese. Foods.

[77] Okur Ö.D. (2021). An Evaluation of the Quality Characteristics of Kefir Fortified with Olive (*Olea Europaea*) Leaf Extract. Br. Food J..

[78] Zandona E., Vranković L., Pedisić S., Vukušić Pavičić T., Dobrinčić A., Marušić Radovčić N., Lisak Jakopović K., Blažić M., Barukčić Jurina I. (2024). Production of Acid and Rennet-Coagulated Cheese Enriched by Olive (*Olea Europaea* L.) Leaf Extract—Determining the Optimal Point of Supplementation and Its Effects on Curd Characteristics. Foods.

[79] Ronca C.L., Duque-Soto C., Samaniego-Sánchez C., Morales-Hernández M.E., Olalla-Herrera M., Lozano-Sánchez J., Giménez Martínez R. (2024). Exploring the Nutritional and Bioactive Potential of Olive Leaf Residues: A Focus on Minerals and Polyphenols in the Context of Spain’s Olive Oil Production. Foods.

[80] Cedola A., Palermo C., Centonze D., Del Nobile M.A., Conte A. (2020). Characterization and Bio-Accessibility Evaluation of Olive Leaf Extract-Enriched “Taralli”. Foods.

[81] El Dessouky Abdel-Aziz M., Samir Darwish M., Mohamed A.H., El-Khateeb A.Y., Hamed S.E. (2020). Potential Activity of Aqueous Fig Leaves Extract, Olive Leaves Extract and Their Mixture as Natural Preservatives to Extend the Shelf Life of Pasteurized Buffalo Milk. Foods.

[82] Nardella S., Conte A., Del Nobile M.A. (2022). State-of-Art on the Recycling of By-Products from Fruits and Vegetables of Mediterranean Countries to Prolong Food Shelf Life. Foods.

[83] Soutelino M.E.M., de Oliveira Silva A.C., Rocha R.d.S. (2024). Natural Antimicrobials in Dairy Products: Benefits, Challenges, and Future Trends. Antibiotics.

[84] Palmeri R., Parafati L., Trippa D., Siracusa L., Arena E., Restuccia C., Fallico B. (2019). Addition of Olive Leaf Extract (OLE) for Producing Fortified Fresh Pasteurized Milk with an Extended Shelf Life. Antioxidants.

[85] Farrag A., Zahran H., Al-Okaby M., El-Sheikh M., Soliman T. (2020). Physicochemical Properties of White Soft Cheese Supplemented With Encapsulated Olive Phenolic Compounds. Egypt. J. Chem..

[86] Han J., Britten M., St-Gelais D., Champagne C.P., Fustier P., Salmieri S., Lacroix M. (2011). Effect of Polyphenolic Ingredients on Physical Characteristics of Cheese. Food Res. Int..

[87] Silva D.F.d., Matumoto-Pintro P.T., Bazinet L., Couillard C., Britten M. (2015). Effect of Commercial Grape Extracts on the Cheese-Making Properties of Milk. J. Dairy Sci..

[88] Tarchi I., Koubaa M., Ozogul F., Bouaziz M., Aït-Kaddour A. (2025). Influence of Olive Leaf Extract on the Physicochemical Properties of Yogurts Made from Cow, Sheep, and Goat Milk. Food Biosci..

[89] Tarchi I., Bouaziz M., Bhat Z., Aït-Kaddour A. (2025). Effect of Encapsulated Olive Leaf Extract on the Physicochemical, Rheological and Antioxidant Properties of Cantal-Type Cheese. Appl. Food Res..

[90] Tosif M.M., Najda A., Bains A., Krishna T.C., Chawla P., Dyduch-Siemińska M., Klepacka J., Kaushik R. (2021). A Comprehensive Review on the Interaction of Milk Protein Concentrates with Plant-Based Polyphenolics. Int. J. Mol. Sci..

[91] Kieserling H., de Bruijn W.J.C., Keppler J., Yang J., Sagu S.T., Güterbock D., Rawel H., Schwarz K., Vincken J.-P., Schieber A. (2024). Protein–Phenolic Interactions and Reactions: Discrepancies, Challenges, and Opportunities. Compr. Rev. Food Sci. Food Saf..

[92] Everette J.D., Bryant Q.M., Green A.M., Abbey Y.A., Wangila G.W., Walker R.B. (2010). Thorough Study of Reactivity of Various Compound Classes toward the Folin−Ciocalteu Reagent. J. Agric. Food Chem..

[93] van de Langerijt T.M., O’Mahony J.A., Crowley S.V. (2023). Structural, Binding and Functional Properties of Milk Protein-Polyphenol Systems: A Review. Molecules.

[94] Yildirim-Elikoglu S., Erdem Y.K. (2018). Interactions between Milk Proteins and Polyphenols: Binding Mechanisms, Related Changes, and the Future Trends in the Dairy Industry. Food Rev. Int..

[95] Horne D.S., Lucey J.A., McSweeney P.L.H., Fox P.F., Cotter P.D., Everett D.W. (2017). Chapter 5—Rennet-Induced Coagulation of Milk. Cheese.

[96] Ma B., Al-Wraikat M., Shu Q., Yang X., Liu Y. (2024). An Overview of Interactions between Goat Milk Casein and Other Food Components: Polysaccharides, Polyphenols, and Metal Ions. Foods.

[97] Lucey J.A., Johnson M.E., Horne D.S. (2003). Invited Review: Perspectives on the Basis of the Rheology and Texture Properties of Cheese. J. Dairy Sci..

[98] Smith J.R., Hindmarsh J.P., Carr A.J., Golding M.D., Reid D. (2017). Molecular Drivers of Structural Development in Mozzarella Cheese. J. Food Eng..

[99] Milovanovic B., Djekic I., Miocinovic J., Djordjevic V., Lorenzo J.M., Barba F.J., Mörlein D., Tomasevic I. (2020). What Is the Color of Milk and Dairy Products and How Is It Measured?. Foods.

[100] Albay Z. (2022). The Effect of Inulin Addition on Probiotic Bacteria Viability and Volatile Components in Low Fat Probiotic Tulum Cheese. Mljekarstvo.

[101] Hegab O.W., Abdel-Latif E.F., Zaki H., Moawad A. (2021). Fundamental role of *Lactobacillus plantarum* and inulin in improving safety and quality of Karish cheese. Open Vet. J..

[102] Laukova A., Chrastinová Ľ., Placha I., Fockova V., Zábolyová N., Bino E., Gresakova L., Žitňan R., Formelová Z., Ščerbová J. (2024). Dairy-Derived and Bacteriocin-Producing Strain *Lactiplantibacillus* Plantarum LP17L/1: An Assessment of Its Safety and Effect Using Broiler Rabbits. Front. Biosci.-Elite.

[103] Antonino C., Faccia M., Natrella G., Perla C., Caponio G.R., De Angelis M. (2026). Effect of *Lactiplantibacillus Plantarum* and *Carnobacterium* spp. on the Sensory Quality of Burrata Cheese during Storage: A Multi-Technique Study. Food Res. Int..

[104] Vasconcelos D.K.M., de Souza E.L., Viana M.G.S., Campos M.I.F., de Medeiros L.L., Olegário L.S., de Sousa Galvão M., dos Santos K.M.O., do Egito A.S., Madruga M.S. (2025). Supplementation with Lactiplantibacillus Plantarum CNPC003 and Pilosocereus Gounellei Flour Enhances the Properties of Goat Cream Cheese. Microorganisms.

[105] Samelis J., Tsanasidou C., Bosnea L., Ntziadima C., Gatzias I., Kakouri A., Pappas D. (2023). Pilot-Scale Production of Traditional Galotyri PDO Cheese from Boiled Ewes’ Milk Fermented with the Aid of Greek Indigenous *Lactococcus Lactis* Subsp. Cremoris Starter and Lactiplantibacillus Plantarum Adjunct Strains. Fermentation.

[106] Ayyash M., Abu-Jdayil B., Hamed F., Shaker R. (2018). Rheological, Textural, Microstructural and Sensory Impact of Exopolysaccharide-Producing Lactobacillus Plantarum Isolated from Camel Milk on Low-Fat Akawi Cheese. LWT.

