# Supplementary material for: Development of Functional Casu Axedu Cheese Enriched with Olive Leaf Phenolics and a Probiotic-Candidate Lactiplantibacillus plantarum Strain
Source: Foods. 2026 Jul 30;15(15):2694. doi: 10.3390/foods15152694 (PMC13465273; doi:10.3390/foods15152694)
Supplement: Supplementary file 1 [file foods-15-02694-s001.zip › foods-4411967-supplementary.pdf]

SUPPLEMENTARY MATERIALS

Table S1. Analytical parameters of HPLC-PDA quantitative method. Data for calibration curves (Calibration range and linearity), limit of detection (LOD), limit of quantification (LOQ) and recovery (%) values are reported for each target phenolic compound dosed by pure standard.

Compound[a]	Calibration range mg/L	linearity R^2	LOD	LOQ	Repeatability [b] (%RSD)	Recovery			
						mg/L	(%)	mg/L	(%)
L-glu	1.0–10,0	0.9949	0.05	0.15	5.1	5.0	89	10.0	95
Verb	0.25-5.0	0.9993	0.04	0.13	3.2	0.5	93	5.0	98
HTyr	0.10-1.0	0.9991	0.03	0.10	4.7	0.5	85	1.0	94
Olup	1.0-20.0	0.9987	0.06	0.17	3.9	5.0	94	20.0	102

[a] L-glu: luteolin-7-O-glucoside, Verb: verbascoside, HTyr: hydroxytyrosol, Olup: oleuropein. [b] Relative standard deviation (RSD) was calculated from six replicate measurements.

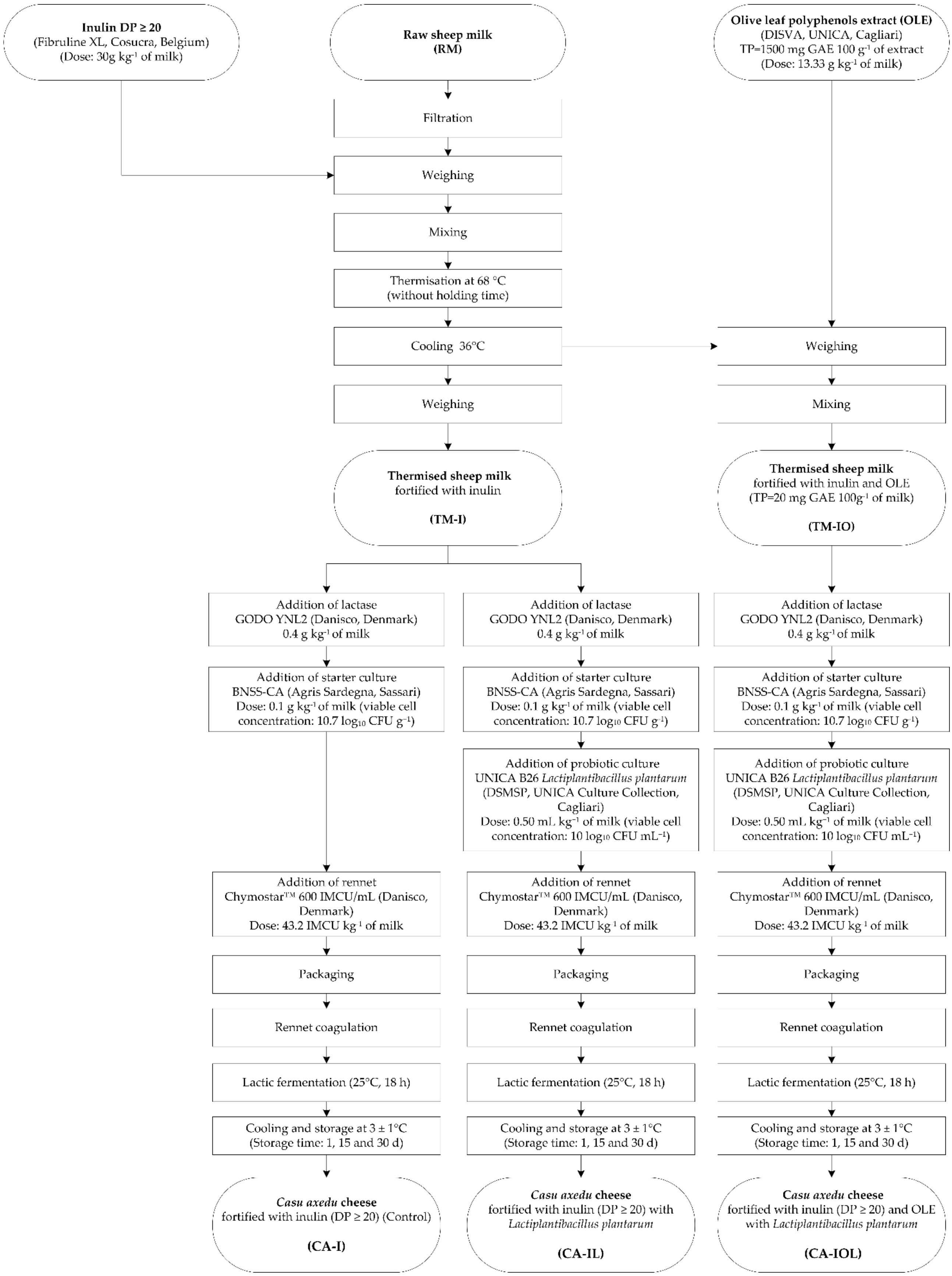

Figure S1. Flow diagram of the pilot-scale production of *Casu Axedu* enriched with inulin and olive leaf extract and inoculated with *Lactiplantibacillus plantarum* strain.

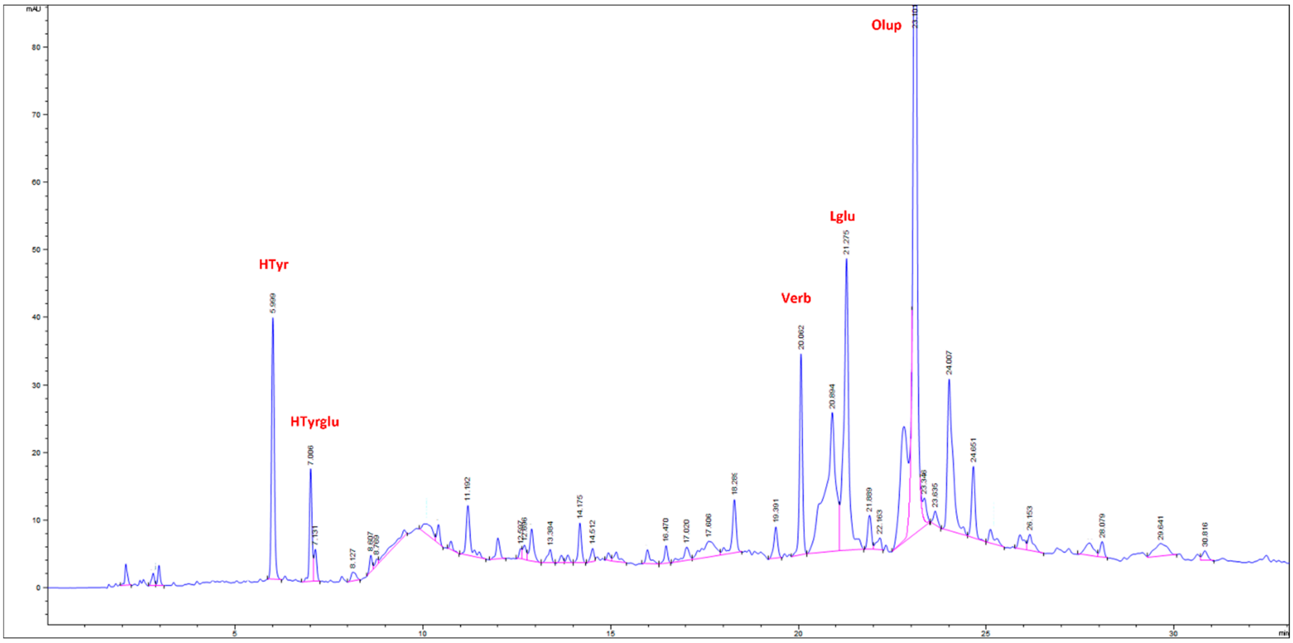

Figure S2. Chromatogram at λ = 280 nm of OLE (diluted 1:200 v/v with 0.22 M H_3PO_4).

L-glu: luteolin-7-*O*-glucoside, Verb: verbascoside, HTyr: hydroxytyrosol, HTyr-glu: hydroxytyrosol glucoside, Olup: oleuropein.

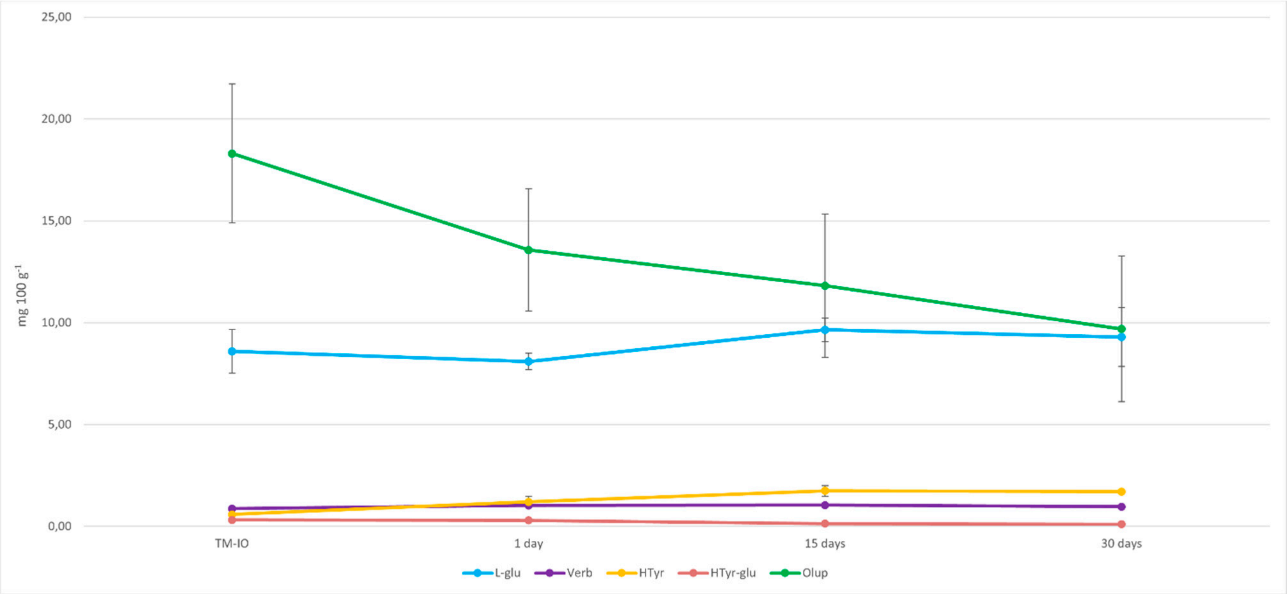

Figure S3. Evolution of target phenolic compounds in fortified *Casu Axedu*.

L-glu: luteolin-7-*O*-glucoside, Verb: verbascoside, HTyr: hydroxytyrosol, HTyr-glu: hydroxytyrosol glucoside, Olup: oleuropein.

Table S2. List of attributes used for Quantitative Descriptive Analysis. For each attribute, the sensory definition, reference standards, and corresponding intensity values (in parentheses) employed for judge calibration are provided.

Attribute	Definition	Standard and Intensity Value
**Colour intensity**	A visual attribute related to the colour tone of the sample, ranging from cream/ivory hues to yellowish-greenish hues	Philadelphia as is (3) - 0.10 mL of olive oil/kg Philadelphia* (7)
**Acidic odour**	Olfactory stimulus perceived via orthonasal way associated with the lactic fermentation of dairy products (e.g. yoghurt)	Philadelphia opened for 24 hours* (3)
**Sour taste**	Elementary taste sensation associated with substances such as lactic acid	0.21 g of lactic acid/kg Philadelphia (2) - 0.42 of lactic acid/kg Philadelphia* (7)
**Sweet taste**	Elementary taste sensation associated with substances such as sucrose	Philadelphia as is* (6)
**Bitter taste**	Elementary taste sensation associated with substances such as caffeine	0.021 g of caffeine/kg Philadelphia (2) - 0.035 g of caffeine/kg Philadelphia* (8)
**Astringency**	A tactile sensation in the mouth characterized by dryness, roughness, and a puckering or tightening feeling of the oral tissues, caused by the precipitation of salivary proteins by certain compounds (e.g., tannins, polyphenols).	0.10 g of tannic acid/kg Philadelphia* (5)
**Sheep milk aroma**	Olfactory stimulus perceived via retronasal way associated with fresh sheep milk	Sheep milk ricotta cheese (5)
**Fresh milk aroma**	Olfactory stimulus perceived via retronasal way associated with fresh milk, which evokes the sweetness of lactose	Partially skimmed UHT milk (5)
**Olive oil aroma**	Olfactory stimulus perceived via retronasal way associated with fresh, fruity and vegetal notes of olive oil	0.10 mL of olive oil/Kg di Philadelphia* (6)
**Acidic aroma**	Olfactory stimulus perceived via retronasal, slightly tangy, associated with the lactic acid fermentation of dairy products, e.g. yoghurt	Philadelphia opened for 24 hours* (3)
**Watery**	A mouthfeel sensation perceived in the oral cavity, resulting from the sample's tendency to release or exude water during evaluation.	Commercial *Casu Axedu* (5)
**Cohesiveness**	A tactile sensation perceived in the oral cavity by placing the sample between the molars, applying pressure, and evaluating the degree of deformation as well as the sample's ability to remain intact prior to fracture and/or disintegration	Panna cotta (3)
**Off-Flavour**	Presence of anomalous sensations, e.g., rancid, oxidized, pungent, etc.	
**Overall sensory quality**	General liking of the analysed product	

*Aqueous solutions were prepared maintaining a fixed water/Philadelphia cheese ratio (143 g/kg)

Table S3. Free fatty acids content and proteolysis indices of *Casu Axedu* enriched with inulin and olive leaf extract and inoculated with *Lactiplantibacillus plantarum* UNICA B26 during storage.

	Formulation factor			Storage time at 3 ± 1 °C			*p*-value[12]		
	**CA-I**[1]	**CA-IL**[2]	**CA-IOL**[3]	**1 day**	**15 days**	**30 days**	**SEM**[11]	FF[13]	S[14]
Free fatty acids									
SCFFA[4]	0.34	0.34	0.35	0.34	0.34	0.35	0.07	ns	ns
MCFFA[5]	0.8	0.9	0.9	0.9	0.8	0.9	0.2	ns	ns
LCFFA[6]	1.0	1.1	1.1	1.1	1.0	1.0	0.2	ns	ns
TFFA[7]	2.2	2.3	2.4	2.3	2.2	2.3	0.3	ns	ns
Proteolysis indices									
SN/TN (%)[8]	27.4	27.8	27.3	23.5^{C}	27.1^{B}	31.9^{A}	0.9	ns	*
TCA-SN/TN (%)[9]	12	13	12	10^{B}	12^{B}	15^{A}	1	ns	*
PTA-SN/TN (%)[10]	5.8	6.2	6.3	5.6^{B}	5.8^{B}	6.9^{A}	0.5	ns	*

[1]**CA-I**, *Casu Axedu* cheese fortified with inulin (DP ≥ 20) (Control); [2]**CA-IL**, *Casu Axedu* cheese fortified with inulin (DP ≥ 20) and *Lactiplantibacillus plantarum* (UNICA B26); [3]**CA-IOL**, *Casu Axedu* cheese fortified with inulin (DP ≥ 20) and OLE (TP=20 mg GAE 100 g^{-1} of milk) and *Lactiplantibacillus plantarum* (UNICA B26); [4]**SCFFA**, short chain free fatty acids; [5]**MCFFA**, medium chain free fatty acids; [6]**LCFFA**, long chain free fatty acids; [7]**TFFA**, total free fatty acids; [8]**SN**, water-soluble nitrogen at pH 4.6; **TN**, total nitrogen; [9]**TCA-SN**, 12% trichloroacetic acid-soluble nitrogen; **TN**, total nitrogen; [10]**PTA-SN**, 10% phosphotungstic acid-soluble nitrogen; **TN**, total nitrogen; [11]**SEM**, standard error of the mean; [12]***p*-value**, ns, no significant difference ($p > 0.05$); *$p < 0.05$; [13]**FF**, formulation factor; [14]**S**, storage time factor at 3 ± 1 °C; different superscript letters within rows indicate significant differences; lowercase letters, indicate significant differences between levels of the formulation factor (FF); uppercase letters, indicate significant differences among levels of the storage time factor (S).

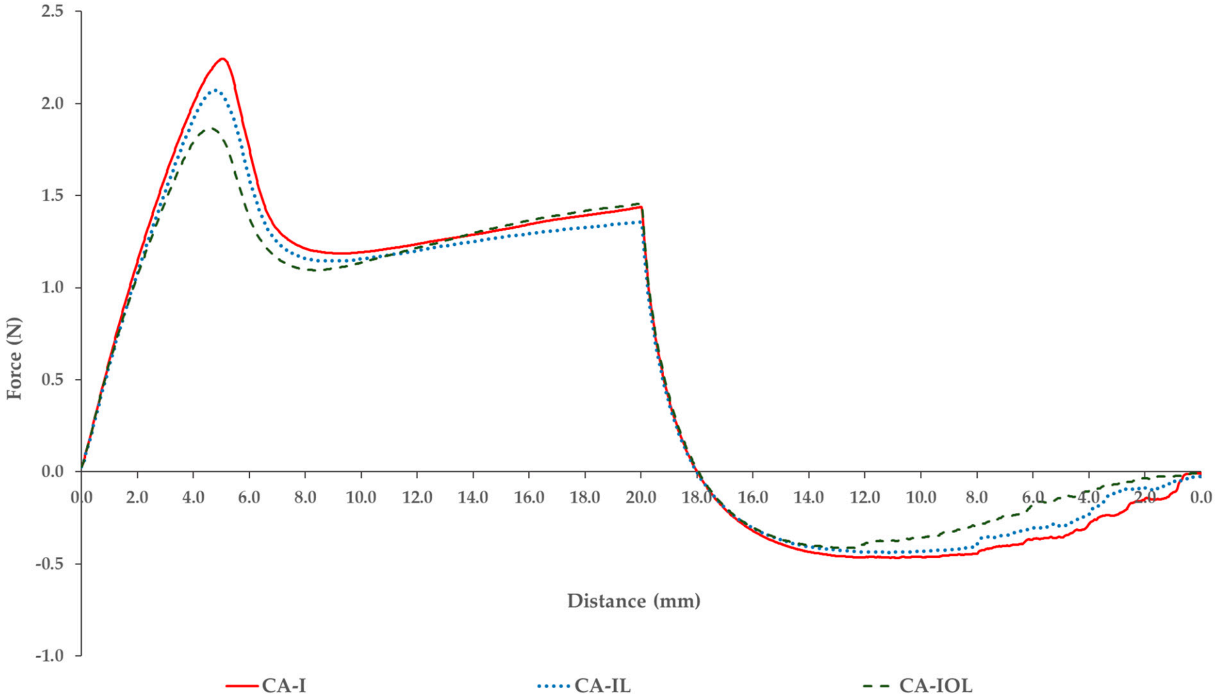

Figure S4. Penetration profile of *Casu Axedu* cheese (**CA-I**, *Casu Axedu* cheese fortified with inulin (DP ≥ 20) (Control); **CA-IL**, *Casu Axedu* cheese fortified with inulin (DP ≥ 20) and *Lactiplantibacillus plantarum* UNICAB26; **CA-IOL**, *Casu Axedu* cheese fortified with inulin (DP ≥ 20), OLE (TP=20 mg GAE 100 g^{-1} of milk) and *Lactiplantibacillus plantarum* UNICA B26.

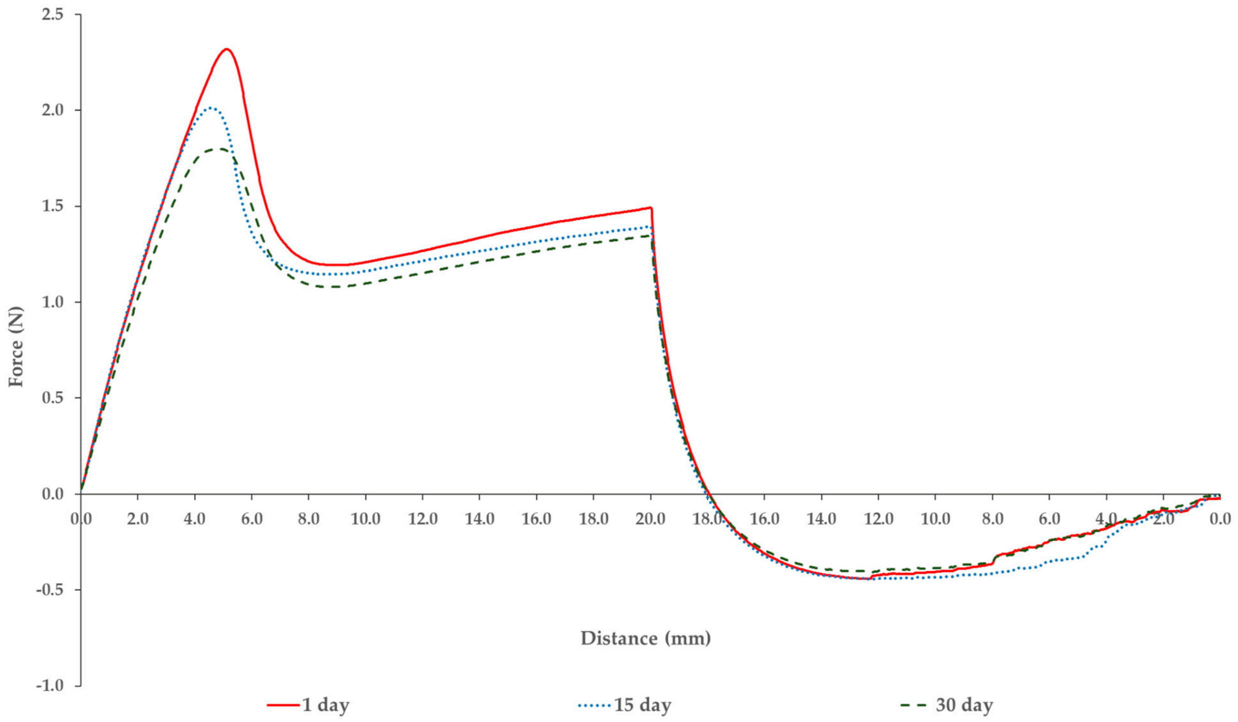

Figure S5. Penetration profile of *Casu Axedu* cheese during storage time at 3 ± 1 °C.

Table S4. Acidification kinetic parameters of *Casu Axedu* enriched with inulin and olive leaf extract and inoculated with *Lactiplantibacillus plantarum* UNICA B26 during fermentation.

	**CA-I**[1]	**CA-IL**[2]	**CA-IOL**[3]	**SEM**[7]	***p*-value**[8]
Vm (mUpH min^{-1})[4]	-6.6	-6.3	-5.8	0.6	ns
Tm (min)[5]	654	678	631	40	ns
pHm (UpH)[6]	5.77	5.64	5.81	0.06	ns

[1]**CA-I**, *Casu Axedu* cheese fortified with inulin (DP ≥ 20) (Control); [2]**CA-IL**, *Casu Axedu* cheese fortified with inulin (DP ≥ 20) and *Lactiplantibacillus plantarum* (UNICA B26); [3]**CA-IOL**, *Casu Axedu* cheese fortified with inulin (DP ≥ 20) and OLE (TP=20 mg GAE 100 g^{-1} of milk) and *Lactiplantibacillus plantarum* (UNICA B26); [4]**Vm (mUpH min^{-1})**, maximum acidification rate; [5]**Tm (min)**, time at which Vm occurred; [6]**pHm (UpH)**, pH at which Vm was reached; [7]**SEM**, standard error of the mean; [8]***p*-value**, ns, no significant difference ($p > 0.05$).

Table S5. RV coefficients among QDA, CATA and EDO datasets and the global MFA configuration.

	**QDA**	**CATA**	**EDO**	**MFA**
**QDA**	1.0000	0.9005	0.7933	0.9461
**CATA**	0.9005	1.0000	0.9031	0.9798
**EDO**	0.7934	0.9031	1.0000	0.9361
**MFA**	0.9461	0.9798	0.9361	1.0000

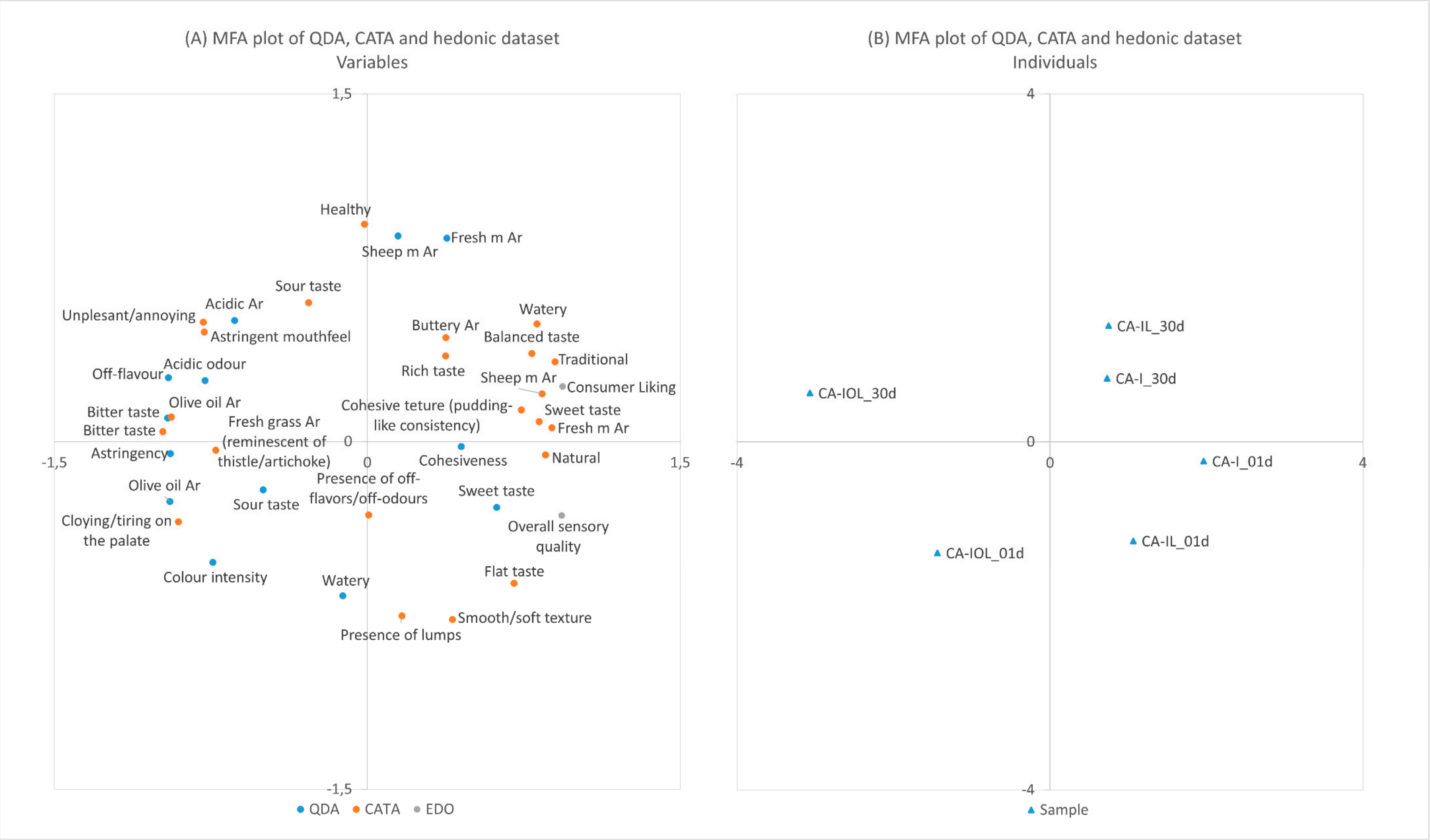
(A) MFA plot of QDA, CATA and hedonic dataset
Variables
1,5
-1,5
0
1,5
-1,5
Healthy
Sheep m Ar
Fresh m Ar
Sour taste
Acidic Ar
Unplesant/annoying
Astringent mouthfeel
Watery
Buttery Ar
Balanced taste
Traditional
Rich taste
Sheep m Ar
Consumer Liking
Off-flavour
Acidic odour
Olive oil Ar
Cohesive teture (pudding-like consistency)
Sweet taste
Fresh m Ar
Bitter taste
Bitter taste
Fresh grass Ar (reminescent of thistle/artichoke)
Astringency
Cohesiveness
Natural
Presence of off-flavors/off-odours
Olive oil Ar
Sour taste
Sweet taste
Cloying/tiring on the palate
Overall sensory quality
Colour intensity
Watery
Flat taste
Smooth/soft texture
Presence of lumps
QDA
CATA
EDO
(B) MFA plot of QDA, CATA and hedonic dataset
Individuals
4
0
-4
4
-4
CA-IL_30d
CA-I_30d
CA-IOL_30d
CA-I_01d
CA-IL_01d
CA-IOL_01d
Sample

Figure S6. Multiple factor analysis (MFA) of QDA, CATA, and hedonic data collected at the storage times common to all datasets. (A) Individual factor map of the experimental cheeses. (B) Variable factor map. Blue labels correspond to QDA attributes, red labels to CATA terms, and green labels to hedonic variables (trained-panel overall sensory quality and consumer overall liking). For readability, "aroma" was abbreviated as "Ar" and "milk" as "m" in the variable labels. Variables projected in the same area are positively associated, whereas variables located on opposite sides are negatively associated. The MFA highlights the opposition between bitter taste, astringency/astringent mouthfeel, and olive oil aroma, and the positive hedonic descriptors associated with liking, overall sensory quality, balanced taste, naturalness, and traditionality.
